# Isthmin-2 is a first-trimester predictor of preeclampsia and fetal growth restriction

**DOI:** 10.1038/s41591-026-04573-6

**Published:** 2026-09-01

**Authors:** Ruoyu Miao, Sungsam Gong, Ulla Sovio, Irving L. M. H. Aye, Giulia Avellino, Jacqueline Guettler, Stephanie N. Rogers, Emma Cook, Ana Toribio, Lina Bergman, Susanne Lager, Anna-Karin Wikström, IMPACT Consortium, Lina Bergman, Susanne Lager, Anna-Karin Wikström, Ylva Carlsson, Stefan R. Hansson, Peter Lindgren, Anders Larsson, Peter Conner, Alice Zancanaro, Marius Kublickas, Anna Sandström, Paul D. W. Kirk, D. Stephen Charnock-Jones, Gordon C. S. Smith

**Affiliations:** 1 Department of Obstetrics and Gynaecology, University of Cambridge; NIHR Cambridge Biomedical Research Centre, Cambridge, UK; 2 The Loke Centre for Trophoblast Research, Department of Physiology, Development and Neuroscience, University of Cambridge, Cambridge, UK; 3 Wellcome - Medical Research Council Cambridge Stem Cell Institute, University of Cambridge, Cambridge, UK; 4 Stratified Medicine Core Laboratory, Department of Genomic Medicine, University of Cambridge, Cambridge, UK; 5 Department of Obstetrics and Gynecology, Institute of Clinical Sciences, University of Gothenburg, Sahlgrenska Academy, Gothenburg, Sweden; 6 Department of Obstetrics and Gynecology, Sahlgrenska University Hospital, Gothenburg, Sweden; 7 Department of Women’s and Children’s Health, Uppsala University, Uppsala, Sweden; 8 Department of Obstetrics and Gynecology, Stellenbosch University, Stellenbosch, South Africa; 9 Wallenberg Center of Molecular and Translational Medicine, University of Gothenburg, Gothenburg, Sweden; 10 Department of Obstetrics and Gynecology, Uppsala University Hospital, Uppsala, Sweden; 11 MRC Biostatistics Unit, University of Cambridge, Cambridge, UK; 12 Department of Obstetrics and Gynecology, Institute of Clinical Sciences Lund, Lund University, Lund, Sweden; 13 Department of Obstetrics and Gynecology, Skåne University Hospital Lund/Malmö, Skåne, Sweden; 14 Center for Fetal Medicine, Karolinska University Hospital, Stockholm, Sweden; 15 Department of Women’s and Children’s Health, Karolinska Institutet, Stockholm, Sweden; 16 Department of Medical Sciences, Clinical Chemistry, Uppsala University, Uppsala, Sweden; 17 Division of Obstetrics, Department of Women’s Health, Karolinska University Hospital, Stockholm, Sweden; 18 Clinical Epidemiology Division, Department of Medicine, Karolinska Institutet, Stockholm, Sweden

**Keywords:** Predictive markers, Reproductive biology

## Abstract

Preeclampsia and fetal growth restriction (FGR) are major causes of global morbidity and mortality. Both conditions are associated with impaired invasion of the uterus by extravillous trophoblast (EVT). We performed proteomics in maternal serum obtained at ~12 weeks of gestational age in a prospective pregnancy cohort (Pregnancy Outcome Prediction Study). Here we show that low maternal serum isthmin-2 (ISM2) was the strongest protein signal (out of 2,904) in the first trimester of pregnancy for preeclampsia or FGR. We validated the association in two independent cohorts (Pregnancy Outcome Prediction Study 2 and Improving Maternal Pregnancy And Child ouTcomes study). ISM2 protein and mRNA are almost exclusively produced in the placenta, and, within the placenta, ISM2 mRNA is highly enriched in EVT. Knocking down ISM2 in cultured human trophoblast stem cells profoundly inhibited EVT invasion. Conversely, expressing ISM2 in a cell line lacking endogenous ISM2 (HEK293 cells) promoted migration. We conclude that ISM2 may be causally involved in the early pathophysiology of failed trophoblast invasion and that the protein and its associated pathways are potential targets for the prediction and prevention of preeclampsia and FGR.

## Abstract (web summary)

Through exploration of serum proteomics from pregnancy cohorts, Miao et al. have shown that ISM2 is involved in extravillous trophoblast invasion and that lower levels in early pregnancy may indicate increased risk of preeclampsia and fetal growth restriction.

## Main

Preeclampsia (PE) and fetal growth restriction (FGR) are major causes of maternal and perinatal morbidity and mortality and are thought to be determined by placental dysfunction^1^. It is generally accepted that failure of deep invasion of maternal uterine resistance blood vessels by extravillous trophoblast (EVT) is a key early feature of both conditions^2^. However, there is a lack of mechanistic understanding of why EVT invasion fails. Studying the pathophysiology of failed trophoblast invasion has been problematic. First, humans have the deepest trophoblast invasion of any placental mammal^3^. By contrast, the commonly used mouse model has shallow trophoblast invasion and has, therefore, limited utility for studying failure of deep EVT invasion. Second, for many years, in vitro models of human EVT were limited by the cell lines available, many of which lacked key phenotypic features of normal EVT^4^. In the past decade, human trophoblast stem cell and placental organoid models have been developed^5–7^, which provide improved tools for the assessment of EVT function in vitro. In the present study, we applied proximity extension assay (PEA) proteomics to three prospective cohort studies of pregnant women with first-trimester serum samples, using one cohort for discovery and two for validation. Having identified and validated the strongest first-trimester protein signal for the subsequent experience of PE and FGR, we then evaluated the effects of knockdown in human trophoblast stem cells (hTSCs) and placental organoids and of overexpression in a cell line lacking endogenous isthmin-2 (ISM2).

## Identification of disease-associated proteins

**Fig. 1:**
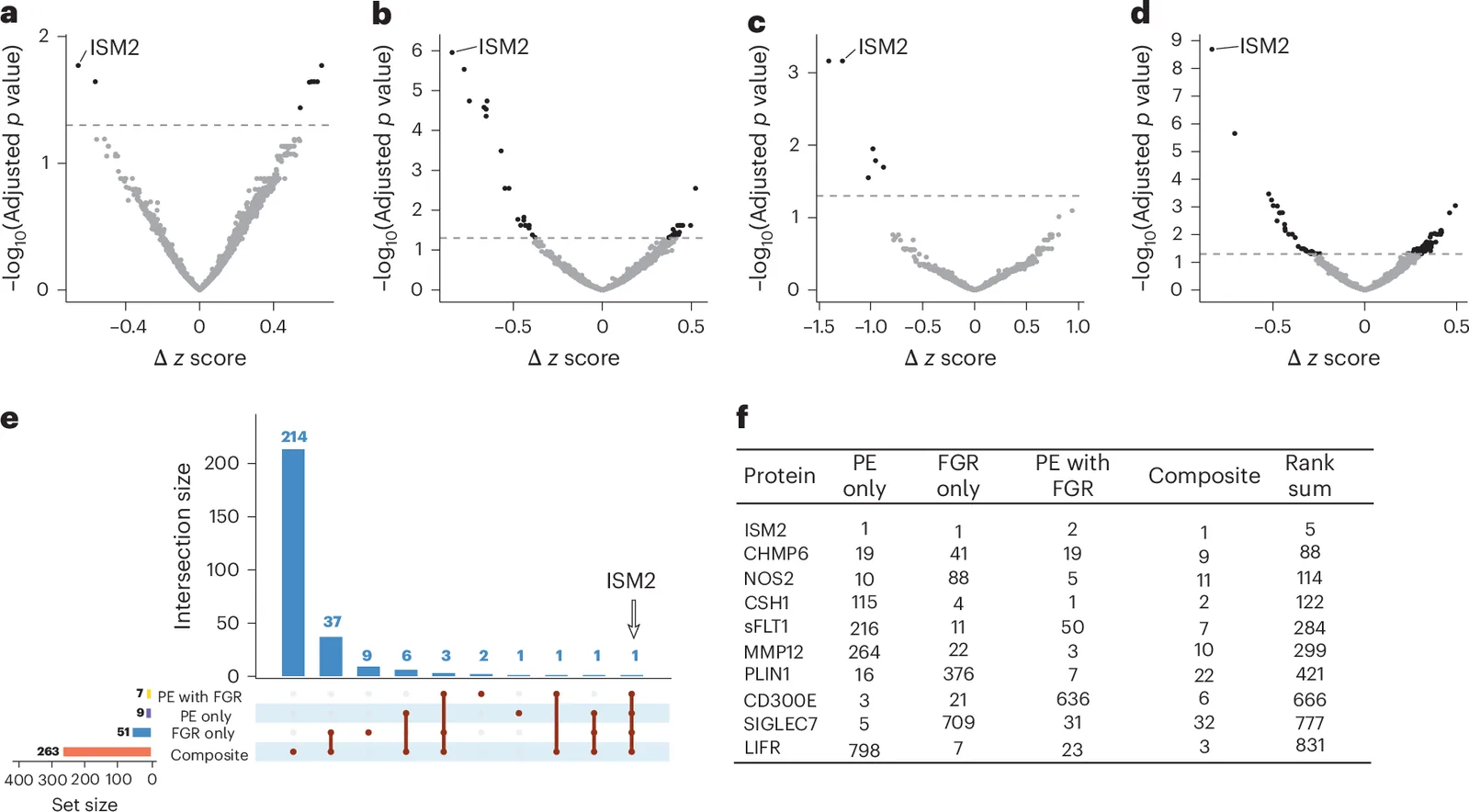
DAPs at 12 wkGA for PE and FGR in POPS. **a**–**d**, Volcano plots for PE only (**a**), FGR only (**b**), PE with FGR (**c**) and a composite of any of the previous three outcomes (**d**). The numbers of control and case samples were 242 and 72 for PE only, 242 and 126 for FGR only, 242 and 25 for PE with FGR, and 242 and 223 for the composite outcome, respectively. The difference between cases and controls (*x* axis) was calculated using the difference in mean NPX (log_2_-transformed protein expression) GA-adjusted *z* scores between cases and controls, and the *p* values were calculated using logistic regression. The dashed line represents *p* = 0.05, corrected for false discovery (5%) using the Benjamini–Hochberg method. **e**, Upset plot of the overlaps between DAPs across the four outcomes. **f**, Table of the ranks for each outcome and the sum of the ranks for the four outcomes for the top ten proteins, in which each individual rank was calculated by *p* value (calculated using logistic regression), that is, the lowest *p* values ranked the highest. Source data

**Extended Data Fig. 1:**
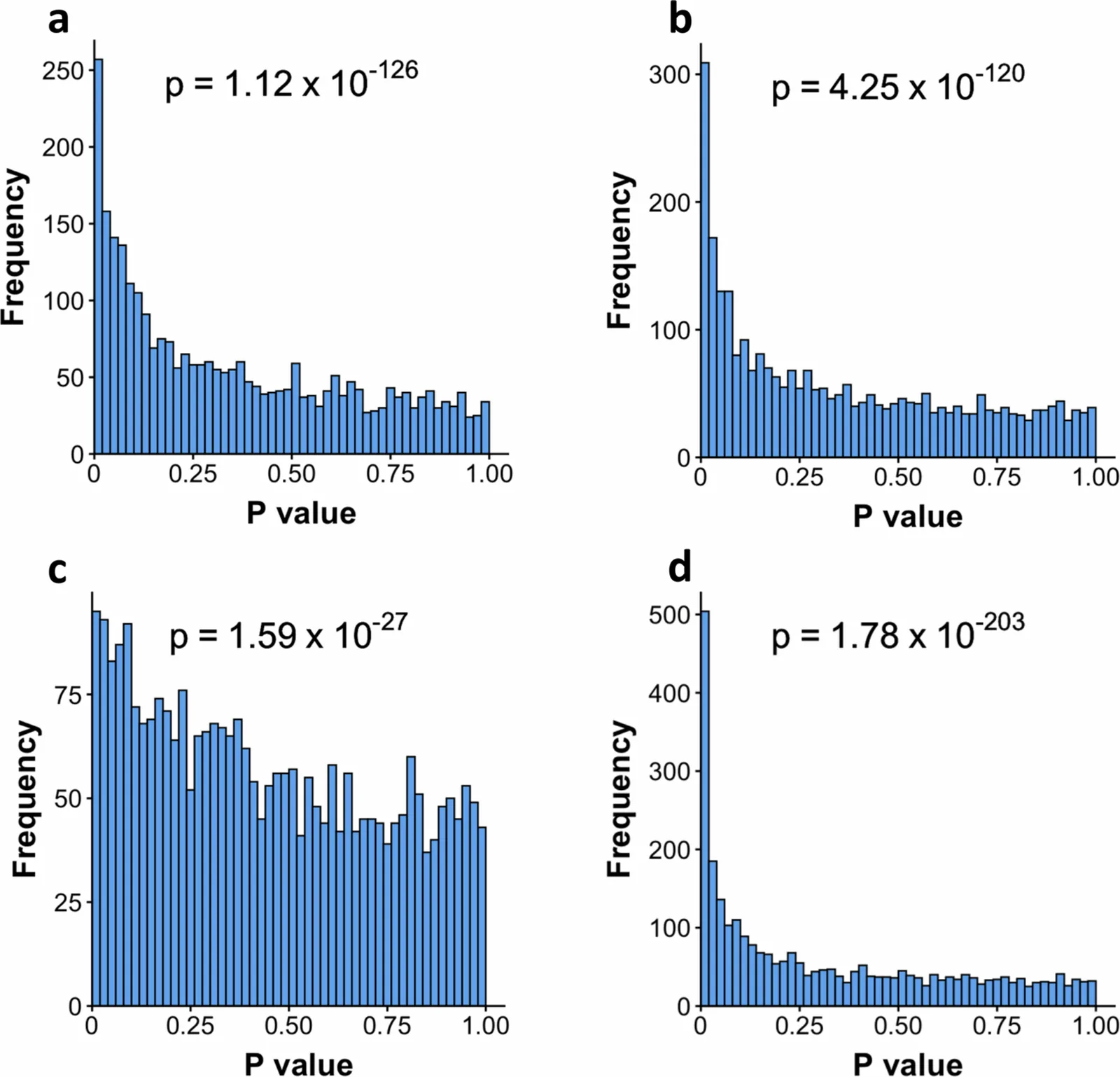
Test for excess of small p values across the four outcomes in the POPS cohort. Plots of p value frequency distributions across the 2,904 proteins studied at 12wkGA for **a**. PE only, **b**. FGR only, **c**. PE with FGR, and **d**. a composite of any of the three outcomes. The p value is for the null hypothesis that the distribution of p values follows a uniform distribution (does not differ across the range) and is calculated using the Kolmogorov-Smirnov test. The numbers of control and case samples were 242 and 72 for PE only, 242 and 126 for FGR only, 242 and 25 for PE with FGR, and 242 and 223 for composite, respectively. Source data

We analyzed samples from the Pregnancy Outcome Prediction Study (POPS)^8,9^, a prospective cohort study of 4,512 unselected nulliparous women with a singleton pregnancy. Blood was obtained at the first visit, which occurred at a mean of 12.7 weeks of gestational age (wkGA), and further blood samples were obtained at ~20 wkGA, ~28 wkGA and ~36 wkGA. We compared proteomic data using a case–cohort study design in POPS, described in detail elsewhere^10,11^. The advantages of a case–cohort design have been described elsewhere^12^, and the large (29,333 participants) European Prospective Investigation into Cancer and Nutrition–Cardiovascular Disease study exemplifies such a design^13^. The characteristics of the patients studied are tabulated (Supplementary Table 1) by their subsequent experience of complications. We quantified the levels of 2,904 proteins using the Olink Explore assay and compared women who subsequently went on to experience complications with a random comparison group. We studied four outcomes: PE without FGR (PE only), FGR without PE (FGR only), the combination of PE and FGR (PE with FGR), and a composite of any of the previous three outcomes. We identified a statistically significant excess of low *p* values for all four outcomes (Extended Data Fig. 1). Volcano plots demonstrated that several proteins at 12 wkGA were significantly associated with the subsequent risk of all four disease outcomes after 5% false discovery rate correction (Fig. 1a–d). The disease-associated proteins (DAPs) are tabulated (Supplementary Tables 2–5). Of the 2,904 proteins analyzed, ISM2 was the only protein that was associated with all four outcomes (Fig. 1e). We then quantified disease association using the *p* value calculated by logistic regression and ranked the 2,904 proteins (rank one being the smallest *p* value) for each of the four outcomes. When we summed the ranks for all four outcomes and ordered the 2,904 proteins in order of the sum of ranks (Fig. 1f), ISM2 was the protein with the strongest disease association, being the top ranked for three outcomes and second ranked for the fourth outcome, yielding a rank sum of 5. The next highest protein had a rank sum of 88.

## Low ISM2 levels are strongly associated with pregnancy disorders during the first half of gestation

**Fig. 2:**
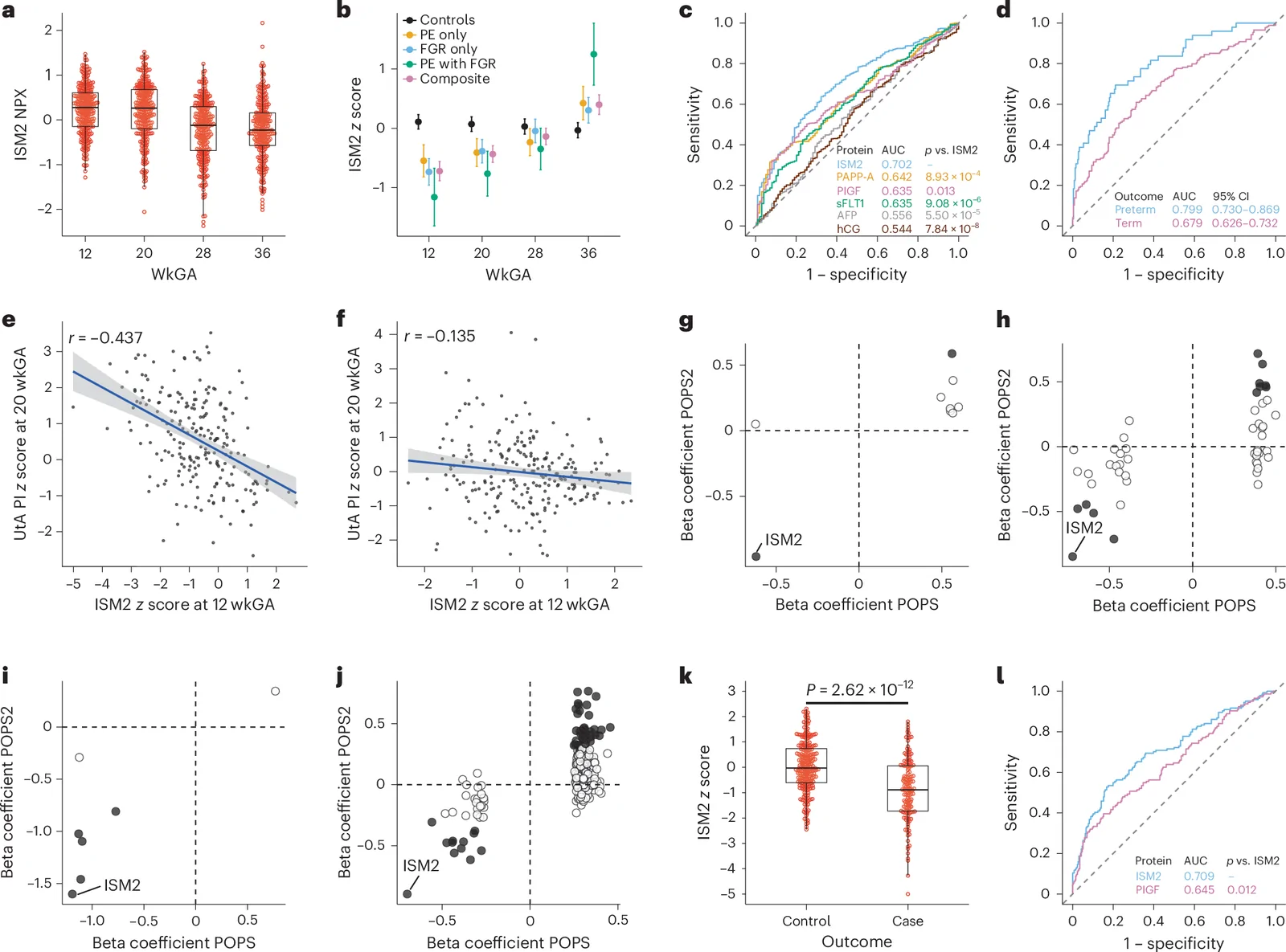
The association between ISM2 and PE and FGR. **a**, Scatter plot of ISM2 values (quantified by arbitrary NPX units) at 12 wkGA (*n* = 301), 20 wkGA (*n* = 305), 28 wkGA (*n* = 303) and 36 wkGA (*n* = 291) in the random comparison group in POPS. Median and interquartile range (IQR, box) are plotted; the whiskers extend to 1.5 × IQR. **b**, The mean and 95% CI of the GA-corrected *z* score (based on the distribution in the whole comparison group) for the four outcomes and the comparison group excluding cases (controls) in POPS. Sample sizes (controls, cases) were as follows: for PE only: 12 wkGA (242, 72), 20 wkGA (249, 72), 28 wkGA (245, 74) and 36 wkGA (239, 62); for FGR only: 12 wkGA (242, 126), 20 wkGA (249, 125), 28 wkGA (245, 123) and 36 wkGA (239, 106); for PE with FGR: 12 wkGA (242, 25), 20 wkGA (249, 24), 28 wkGA (245, 20) and 36 wkGA (239, 10); and for composite: 12 wkGA (242, 223), 20 wkGA (249, 221), 28 wkGA (245, 217) and 36 wkGA (239, 178). **c**, ROC curve plots of ISM2, using the Olink assay, were compared with PAPPA, PlGF, sFLT1, hCG and AFP measured using a clinical platform in the same samples. The performance of the five placental biomarkers was compared with that of ISM2 using the DeLong test. The number of control and case samples (composite outcome) was 242 and 223, respectively. **d**, ROC curve plot for ISM2 at 12 wkGA for the composite outcomes in POPS (controls, cases), comparing cases resulting in preterm birth (242, 49) and term birth (233, 174). **e**,**f**, Plot of the ISM2 GA-adjusted *z* score at 12 wkGA (*x* axis) and the GA-adjusted *z* score for mean uterine artery (UtA) pulsatility index at 20 wkGA (*y* axis) in cases of the composite outcome (*n* = 218) (**e**) and controls (*n* = 233) from POPS (**f**) (*r* is the Pearson correlation coefficient; shading indicates 95% confidence band). **g**–**j**, Plots of the coefficients at 12 wkGA from logistic regression of DAPs identified in POPS, with the POPS coefficients on the *x* axis and the POPS2 coefficients on the *y* axis for PE only (**g**), FGR only (**h**), PE with FGR (**i**) and a composite of any of the previous three outcomes (**j**). Black data points represent *p* < 0.05 (not false discovery rate corrected) in POPS2, and white data points represent *p* ≥ 0.05 comparing cases and controls in POPS2. **k**, Box plot of GA-adjusted *z* score of ISM2 at around 12 wkGA, using the Olink Focus panel, for samples from the IMPACT study for complicated PE (256 controls, 144 cases) analyzed using a two-sided Welch’s *t*-test. Median and IQR (box) are plotted; the whiskers extend to 1.5 × IQR. **l**, ROC curve plots of ISM2 and PlGF, using the Olink Focus panel, for samples from the IMPACT study for complicated PE (256 controls, 144 cases). The performance of the PlGF assay was compared with that of ISM2 using the DeLong test. Source data

The analysis of ISM2 levels, expressed in arbitrary Normalized Protein eXpression (NPX) units, in the random comparison group throughout pregnancy demonstrated stable levels between 12 wkGA and 20 wkGA, then a decline from 20 wkGA to 36 wkGA (Fig. 2a). A quantitative assay of ISM2 levels in a subset of nine samples from ~20 wkGA demonstrated a mean concentration of 262 ng ml^−1^ (s.d. 130). Comparison of levels between cases and controls (expressed as GA-adjusted *z* scores) demonstrated that the biggest differences in ISM2 were observed at 12 wkGA (Fig. 2b). Levels remained lower in all case types at 20 wkGA. However, levels did not differ between the cases and controls at 28 wkGA, and at 36 wkGA, the association was reversed, with ISM2 levels being higher in the cases. When we benchmarked the association against existing biomarkers, the area under the receiver operating characteristic (ROC) curve (AUC, a clinical measure of prognostic discrimination) of the composite outcome was statistically significantly higher for ISM2 measured using the Olink assay than for any of five placental biomarkers measured in the same samples at 12 wkGA using a clinical analysis platform (Fig. 2c). The AUC for ISM2 at 12 wkGA was higher for cases of the composite outcome resulting in preterm birth than for those resulting in term birth (Fig. 2d). PE and FGR associated with preterm birth are more strongly associated with failure of EVT invasion^14^. Finally, there was a striking negative association between ISM2 levels at 12 wkGA and ultrasonic markers of resistance to flow in the mother’s uterine arteries at 20 wkGA in the cases but not in the controls (*p* = 0.0015 for interaction; Fig. 2e,f).

## External validation of ISM2

**Extended Data Fig. 2:**
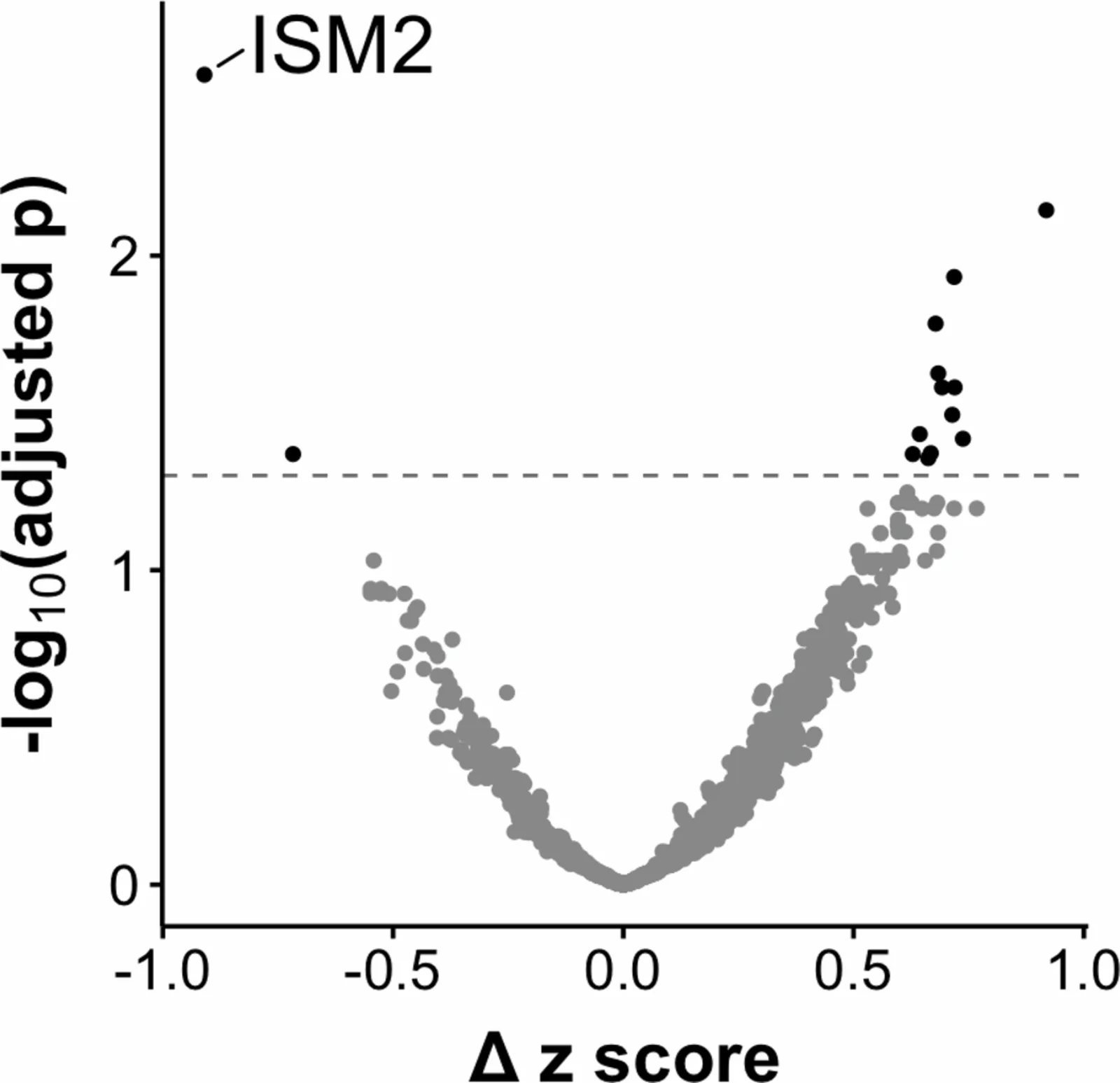
Volcano plot for the composite outcome in the POPS2 validation cohort. The numbers of control and case samples were 85 and 78, respectively. The difference between cases and controls at 12wkGA (x axis) was calculated using the difference in mean NPX (log_2_-transformed protein expression) gestational age adjusted z scores between cases and controls and the p values were calculated using logistic regression. The dotted line represents p = 0.05, corrected for false discovery (5%) using the Benjamini-Hochberg method. Source data

**Extended Data Fig. 3:**
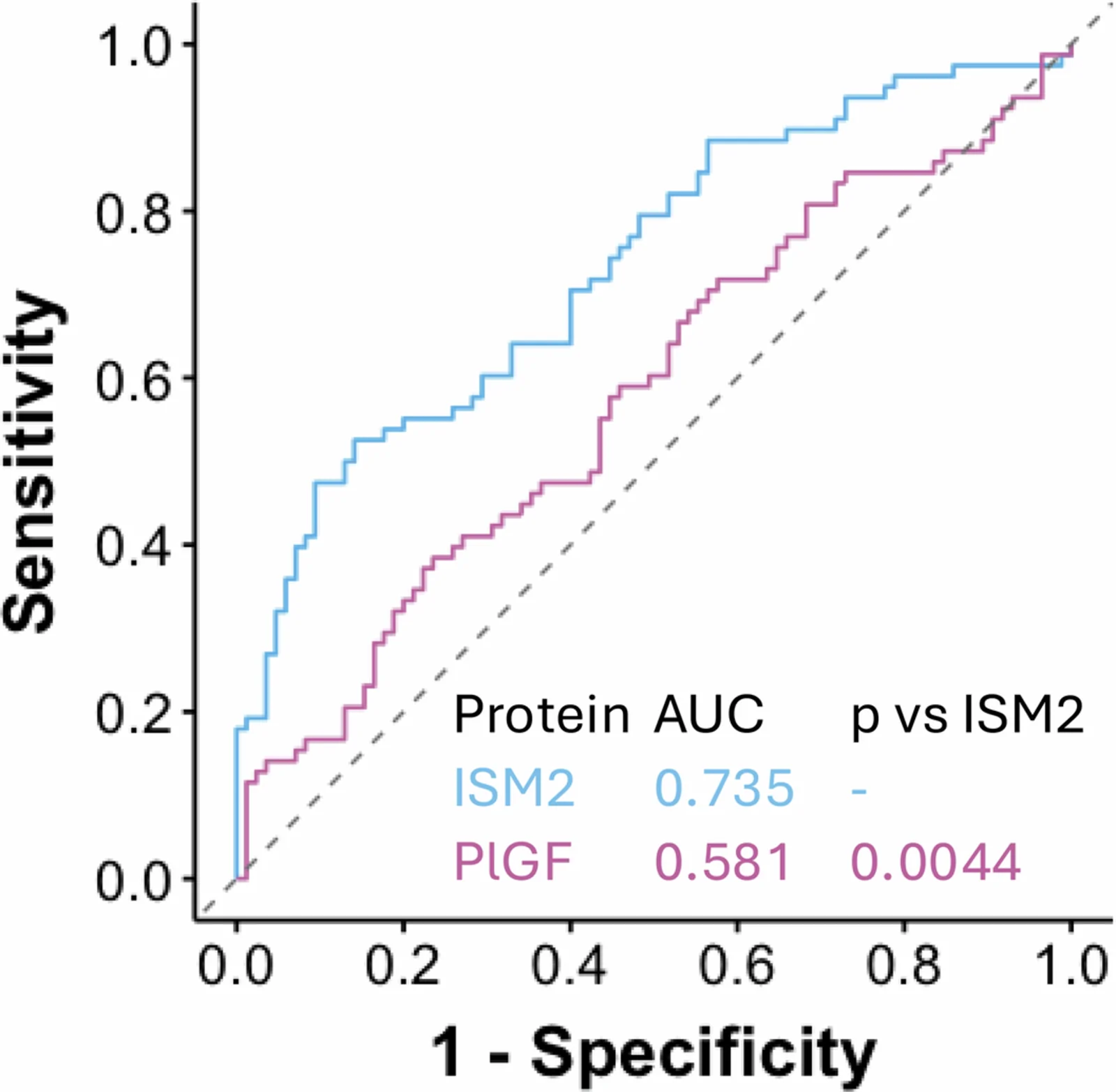
ROC curve plots of ISM2 and PlGF in the POPS2 validation cohort. ISM2 and PlGF were measured using the Olink Explore panel in samples from the POPS2 cohort. The number of control and case (composite outcome) samples was 85 and 78, respectively. The performance of the PlGF assay was compared to that of ISM2 using the DeLong test. Source data

We analyzed samples from the first 2,595 patients with outcome data from the ongoing POPS2 cohort^15^. This is also a prospective cohort study of unselected nulliparous women with a singleton pregnancy, and the schedule of blood sampling is identical to that of POPS. The analysis of proteomic data again used a case–cohort study design. The characteristics of the patients included are tabulated (Supplementary Table 6). When we plotted the beta coefficients obtained from logistic regression for the DAPs identified in the POPS cohort, the beta coefficients for ISM2 across the four outcomes were highly consistent between the two cohorts (Fig. 2g–j). When proteins were ranked by the logistic regression *p* value, ISM2 also had the lowest sum of ranks across the four outcomes in the validation cohort (Supplementary Table 7). Similarly, a volcano plot of the composite outcome demonstrated ISM2 as the strongest signal in POPS2 (Extended Data Fig. 2). We compared the performance of ISM2 with PlGF in the POPS2 cohort (DeLong test), and ISM2 performed significantly better (Extended Data Fig. 3). We performed a further external validation using data and samples from the Improving Maternal Pregnancy And Child ouTcomes (IMPACT) study^16^, a Swedish prospective multicenter cohort of women of mixed parity, which obtained blood between 11 wkGA and 14 wkGA. We measured first-trimester levels of ISM2 and PlGF using a targeted PEA (Olink Focus panel), comparing pregnancies in which the mother subsequently experienced PE complicated by either preterm birth or FGR with a randomly selected subset of controls (see Supplementary Table 8 for demographic details). GA-adjusted *z* scores of ISM2 were significantly lower among cases (*t*-test, *p* = 2.62 × 10⁻^12^; Fig. 2k), and ISM2 had a statistically significantly higher AUC than PlGF (Fig. 2l).

We then used a broad range of statistical and machine-learning methods to develop multivariable models to predict the risk of the composite outcome using DAPs identified in the POPS data (Supplementary Table 9). We trained the models on the POPS data and performed external evaluation in the POPS2 data. A regularized logistic regression model using four protein predictors (CD300E, CHMP6, CSH1 and ISM2) yielded the best out-of-sample prediction with an AUC in POPS2 of 0.740, which was virtually identical to that obtained using ISM2 as the sole predictor (Supplementary Table 9). When we compared the AUC for ISM2 in POPS2 across the different outcomes (Supplementary Table 10), the highest AUC was observed for the combination of PE and FGR (AUC = 0.836, 95% CI 0.687 to 0.986). Previous studies have demonstrated that this outcome is particularly strongly associated with placental dysfunction^17^.

**Extended Data Fig. 4:**
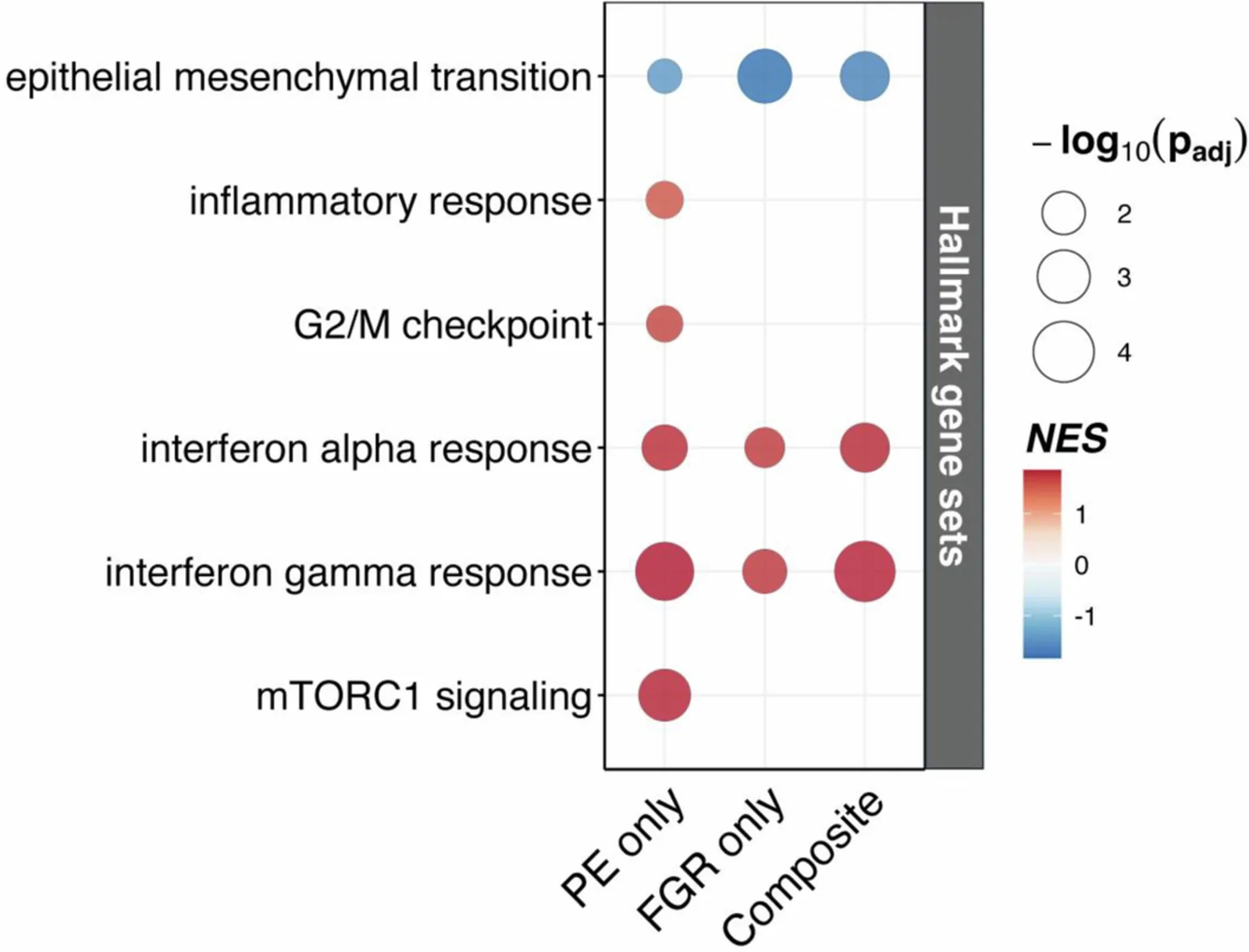
Gene Set Enrichment Analysis (GSEA) of disease associated proteins. Gene set enrichment analysis of the proteins ranked according to their association with PE only, FGR only and the composite outcome using hallmark gene sets from MSigDB. There were no pathways which were significantly enriched when ranked according to association with the PE with FGR group. P_adj_: adjusted p-value; NES: Normalised Enrichment Score. Source data

We performed gene set enrichment analysis (GSEA) for each of the four outcomes using hallmark gene sets from MSigDB^18^. This demonstrated three pathways that were associated with both PE and FGR, namely, epithelial-to-mesenchymal transition, interferon-α (IFNα) response and IFNγ response (Extended Data Fig. 4). This analysis also demonstrated three PE-specific pathways, namely, inflammatory responses, G2/M checkpoint and mTORC1 signaling, but no FGR-specific pathways. There was no pathway significantly associated with the combination of PE and FGR, but this could reflect smaller numbers of this case type.

## ISM2 and trophoblast function

**Fig. 3:**
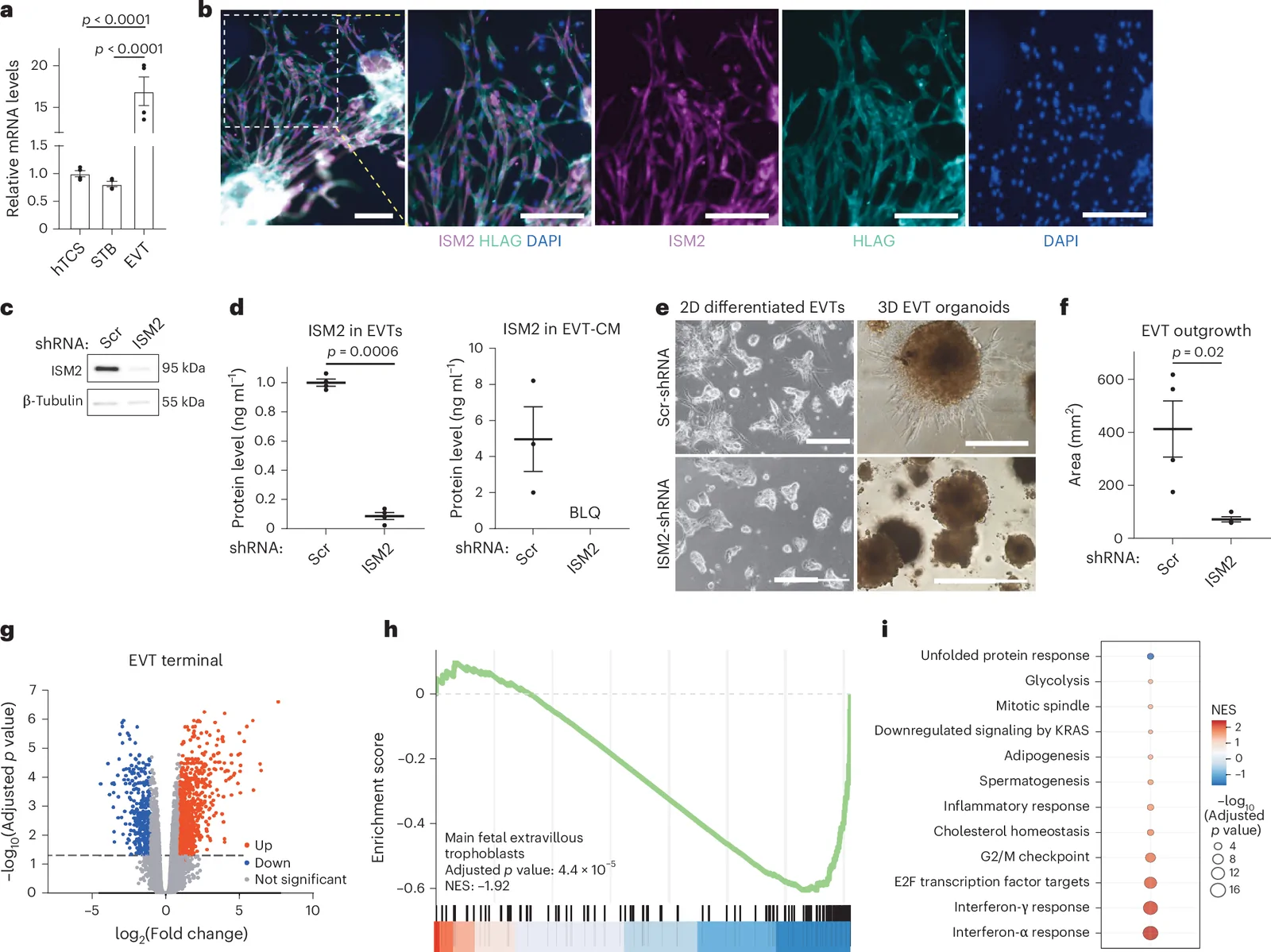
ISM2 regulates EVT differentiation. **a**, *ISM2* mRNA levels in cultured hTSCs, STB and EVTs (*n* = 4 independent cultures; two-sided *p* values are calculated using one-way ANOVA on log-transformed data with Benjamini, Krieger and Yekutieli post-hoc correction; mean ± s.e.m. are plotted). **b**, Co-localization of ISM2 and HLA-G in EVT-differentiated organoids (representative images from three replicates). Scale bars, 500 µm. **c**,**d**, Effects of *ISM2* knockdown on ISM2 levels in EVT cells (immunoblot) (**c**) and ISM2 levels in conditioned media (**d**) (Olink quantitative ISM2 assay; *n* = 3 independent cultures; mean ± s.e.m. are plotted and two-sided *p* values were calculated using Student’s *t*-test). **e**, Bright-field images of hTSCs differentiated into EVTs in two-dimensional (2D) monolayers or three-dimensional (3D) organoids, comparing controls (top) and *ISM2* knockdown (bottom). Scale bars, 300 µm. **f**, Quantification of EVT outgrowths from the trophoblast organoid core (*n* = 4 independent cultures; graphs indicate mean ± s.e.m.; two-sided *p* values were calculated using Student’s *t*-test). **g**, Volcano plot showing DEGs following *ISM2* knockdown in terminally differentiated EVTs. **h**, GSEA of cell-type signatures in Descartes Main Fetal EVT gene set^33^ associated with ISM2 knockdown in terminally differentiated EVTs^18^. **i**, GSEA associated with *ISM2* knockdown in terminally differentiated EVTs using hallmark gene sets from MSigDB. BLQ, below the level of quantification; CM, conditioned media; NES, normalized enrichment score. Source data

**Extended Data Fig. 5:**
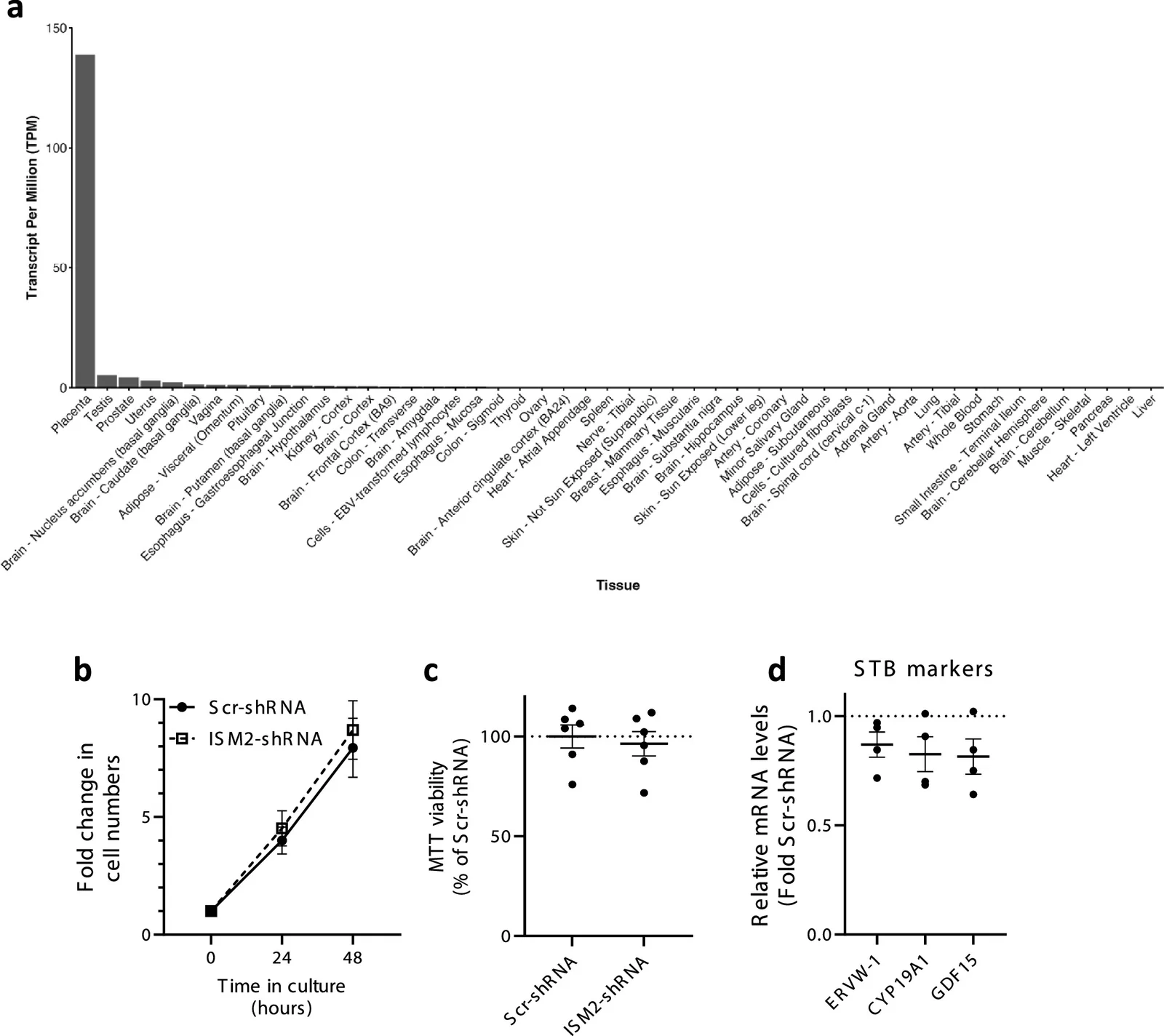
Placental specific expression of ISM2 and its role in regulating trophoblast development. **a**. Re-analysis of a previously described data set^19^ to compare *ISM2* mRNA level in the placenta with 49 tissues reported in the GTEX consortium database^20^. *ISM2* knockdown does not affect **b**. hTSC proliferation, **c**. MTT cell viability or **d**. differentiation into STB. *n* = 4–6, graphs indicate mean ± SEM. Source data

We re-analyzed a previously reported placental RNA sequencing (RNA-seq) dataset^19^, and this demonstrated that placental ISM2 mRNA was ~1,000-fold higher than the median level of tissues in the Genotype–Tissue Expression database^20^ (Extended Data Fig. 5a). We cultured hTSCs and found that differentiation from hTSC to EVT was associated with an ~15-fold increase in ISM2 mRNA, whereas differentiation into syncytiotrophoblast (STB) resulted in ISM2 mRNA levels falling by 20% (Fig. 3a). Consistent with this, dual immunofluorescence staining of trophoblast organoids differentiated into EVTs demonstrated co-localization of ISM2 with the EVT marker HLA-G (Fig. 3b). We next evaluated the effect of knocking down ISM2 in hTSCs using short hairpin RNA (shRNA). ISM2 knockdown did not affect hTSC proliferation, cell viability or differentiation into STB lineage (Extended Data Fig. 5b–d). ISM2 knockdown resulted in >90% reduction in ISM2 in the cells (immunoblot) and reduced ISM2 in the conditioned media from 5 ng ml^−1^ (s.d. 3) to undetectable levels (quantitative Olink Focus panel assay) (Fig. 3c,d). Control hTSC (transduced with scrambled shRNA) differentiated into EVTs and demonstrated epithelial to mesenchyme-like morphological changes, whereas ISM2 knockdown in hTSCs prevented EVT differentiation (Fig. 3e,f). Similarly, when control hTSCs were grown into three-dimensional placental organoids and cultured in Matrigel with EVT differentiation medium to induce differentiation, there was circumferential invasion of the Matrigel by EVTs. This was abolished by ISM2 knockdown (Fig. 3e,f).

## Effect of ISM2 knockdown on the trophoblast transcriptome

**Extended Data Fig. 6:**
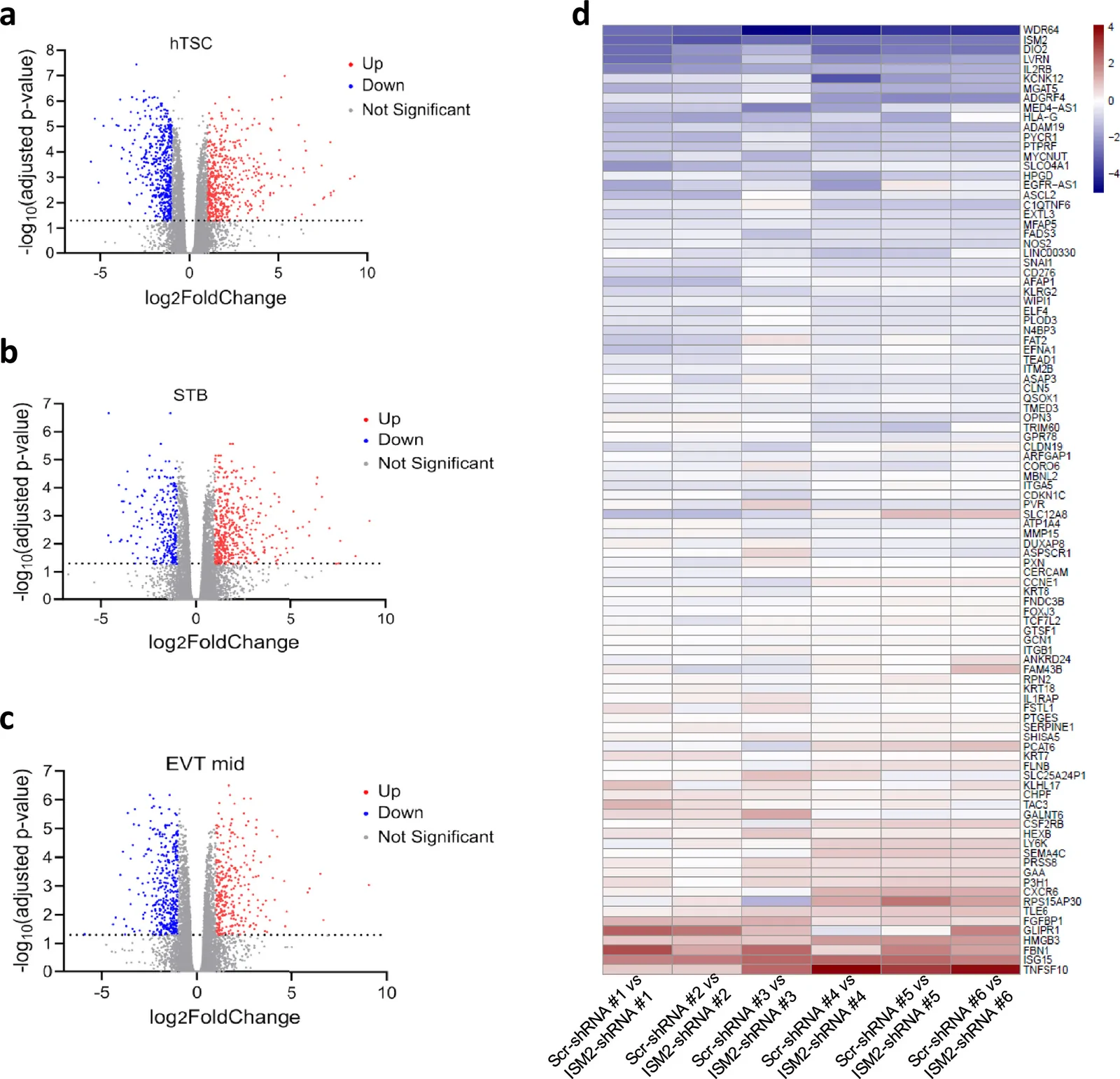
Effects of *ISM2* knockdown on transcriptomes of hTSCs, STB and EVTs (mid-differentiation and terminally differentiated). **a-c**. Volcano plots showing differentially expressed genes (DEGs) following *ISM2* knockdown in (**a**) hTSCs maintained in their stem-state, (**b**) terminally differentiated into STB, or (**c**) mid-differentiation EVTs. **d**. Heatmap showing DEGs in Scr-shRNA versus *ISM2*-shRNA of terminally differentiated EVTs, corresponding to EVT marker gene sets defined in single cell RNA-seq data of human fetal tissues^33^. Source data

**Extended Data Fig. 7:**
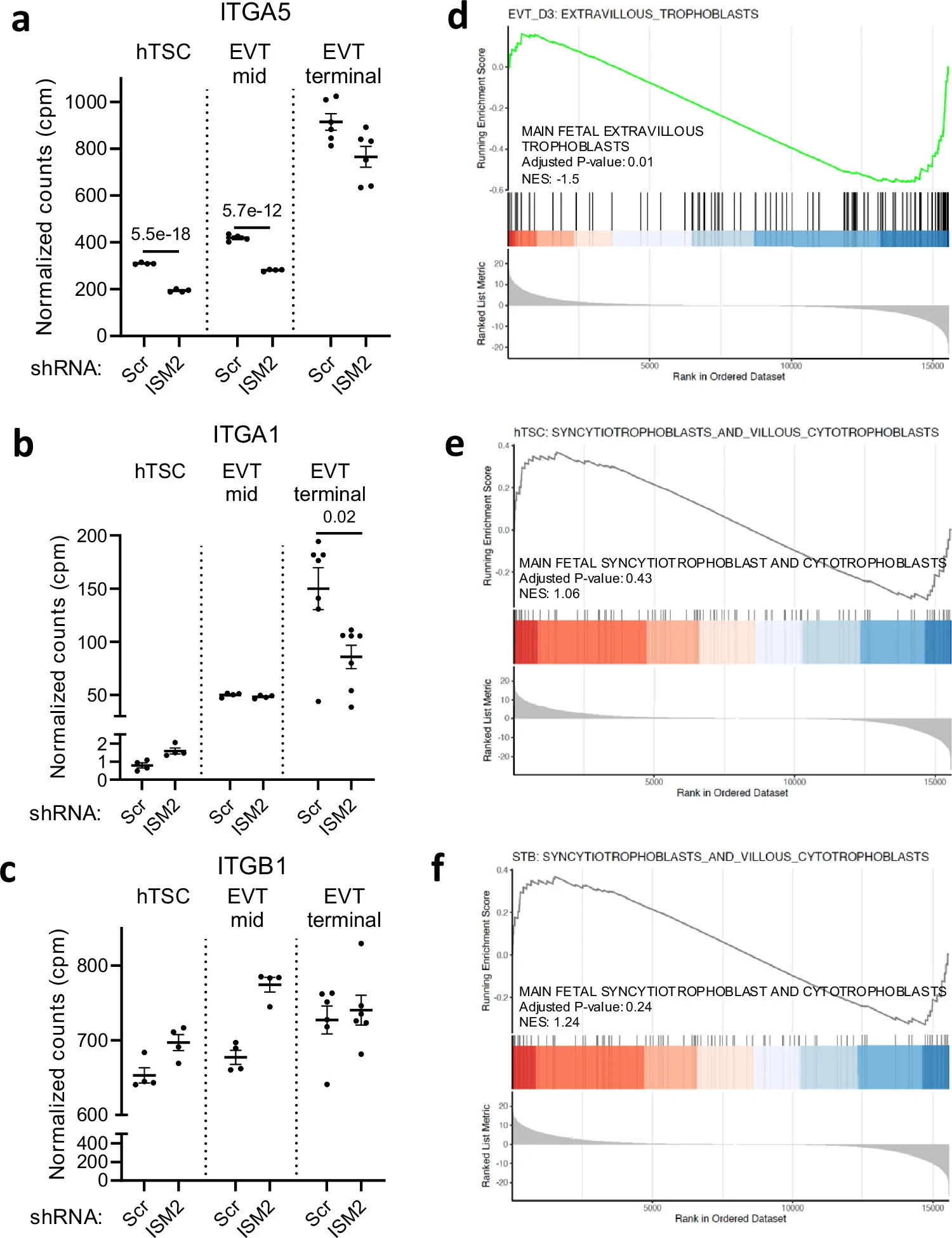
Effects of *ISM2* knockdown on the integrin repertoire associated with EVT differentiation and enrichment of cell type signatures in trophoblast sub-types following *ISM2*-knockdown. **a-c**. Effects of *ISM* knockdown on integrin RNAs (**a**) *ITGA5*, (**b**) *ITGA1* and (**c**) *ITGB1* with hTSCs differentiating into EVTs. *n* = 4–6, graphs indicate mean ± SEM. **d**. Gene set enrichment analysis (GSEA) of cell type signatures in Descartes Main Fetal Extravillous Trophoblast gene set^33^ associated with ISM2 knockdown in mid-differentiation EVTs. **e-f**. GSEA of cell type signatures in Descartes Main Fetal Syncytiotrophoblast and Cytotrophoblasts gene set^33^ associated with *ISM2* knockdown in (**e**) hTSC and (**f**) STB. Source data

We performed RNA-seq analysis comparing *ISM2* knockdown and control trophoblasts in either their stem state (hTSCs) or following differentiation into STB and EVTs (at mid-differentiation point—referred to as EVT mid) or into terminally differentiated EVTs (referred to as EVT terminal). This analysis identified 1,034 differentially expressed genes (DEGs) in hTSCs, 679 DEGs in STB, 696 DEGs in EVT mid and 1,113 DEGs in EVT terminal (edgeR; adjusted *p* value <0.05; absolute fold change >2; Fig. 3g, Extended Data Fig. 6a–c and Supplementary Tables 11–14). Cell-type signature analysis using Descartes main gene sets in the terminally differentiated EVTs highlighted that the fetal EVT set was significantly enriched with downregulated signature genes (adjusted *p* value = 4.4 × 10^−5^; Fig. 3h). This suggests diminished expression of EVT marker genes on *ISM2* knockdown. Consistent with this interpretation, the downregulated genes included several well-characterized EVT marker genes, such as *HLA-G*, *ADAM19*, *ITGA1*, *DIO2* and *SNAI1* (a master regulator of epithelial-to-mesenchymal transition^21^; Extended Data Fig. 6d). Moreover, integrins are associated with trophoblast invasion in vivo^22^, and *ITGA5* was decreased in hTSCs and mid-differentiation EVTs, whereas *ITGA1* was decreased in terminally differentiated EVTs following *ISM2* knockdown (Extended Data Fig. 7a–c). However, the levels of several other EVT signature genes were unchanged by *ISM2* knockdown (for example, *ITGB1* and *KRT7*), indicating that the effect of *ISM2* knockdown was more complex than simply arresting differentiation into EVT. Cell-type signature analysis in mid-differentiation EVTs also demonstrated significant enrichment with fetal EVT in downregulated genes, albeit to a lesser extent (adjusted *p* value = 0.01; Extended Data Fig. 6d). However, the same analysis in hTSCs and STB did not show any significant enrichments with their corresponding cell type (Extended Data Fig. 7d,e). In addition to the downregulated genes, there were 530, 449, 302 and 772 upregulated genes in hTSCs, STB, mid-differentiation EVTs and terminally differentiated EVTs, respectively (edgeR; adjusted *p* value <0.05; absolute fold change >2; Supplementary Tables 11–14). We then focused our analysis on terminally differentiated EVTs because they demonstrated the largest number of DEGs. We performed GSEA associated with *ISM2* knockdown using hallmark gene sets from MSigDB and identified 12 significantly enriched pathways (Fig. 3i). Of these, two were identified in the analysis of PE and FGR DAPs (Extended Data Fig. 4), namely, IFNα response and IFNγ response, and two were also identified in the analysis of PE DAPs, namely, inflammatory response and G2/M checkpoint.

## Effects of exogenous ISM2 and studies in other cell types

**Fig. 4:**
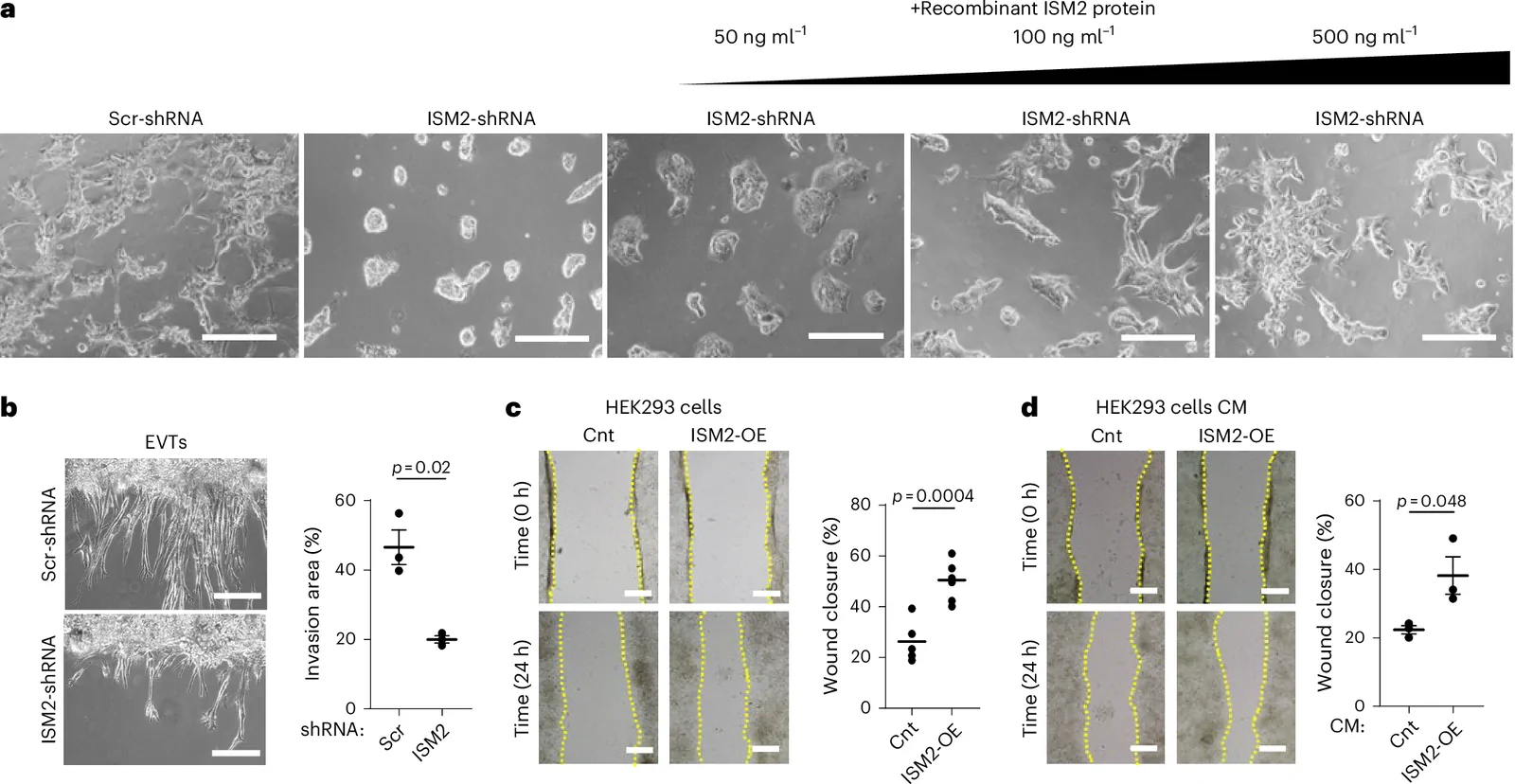
ISM2 regulates cell migration. **a**, The effects of *ISM2* knockdown and the addition of increasing concentrations of recombinant ISM2 on hTSC differentiation into EVTs (representative images from three replicates). Scale bars, 300 µm. **b**, Invasive capacity of EVT transduced with scrambled or *ISM2*-shRNA into Matrigel. Scale bars, 300 µm. **c**,**d**, Wound closure assay in HEK293 cells transduced with control (empty vector) or ISM2 vectors (**c**) and in HEK293 cells incubated with conditioned media from control or *ISM2* overexpressed cells in **c** (**d**). Scale bars, 500 µm. The same fields were imaged for quantification of the scratched (open) areas at times 0 h and 24 h, respectively. In **b** and **d**, *n* = 3; in **c**, *n* = 6. Graphs indicate mean ± s.e.m., and two-sided *p* values were calculated using Student’s *t*-test. OE, overexpression. Source data

**Extended Data Fig. 8:**
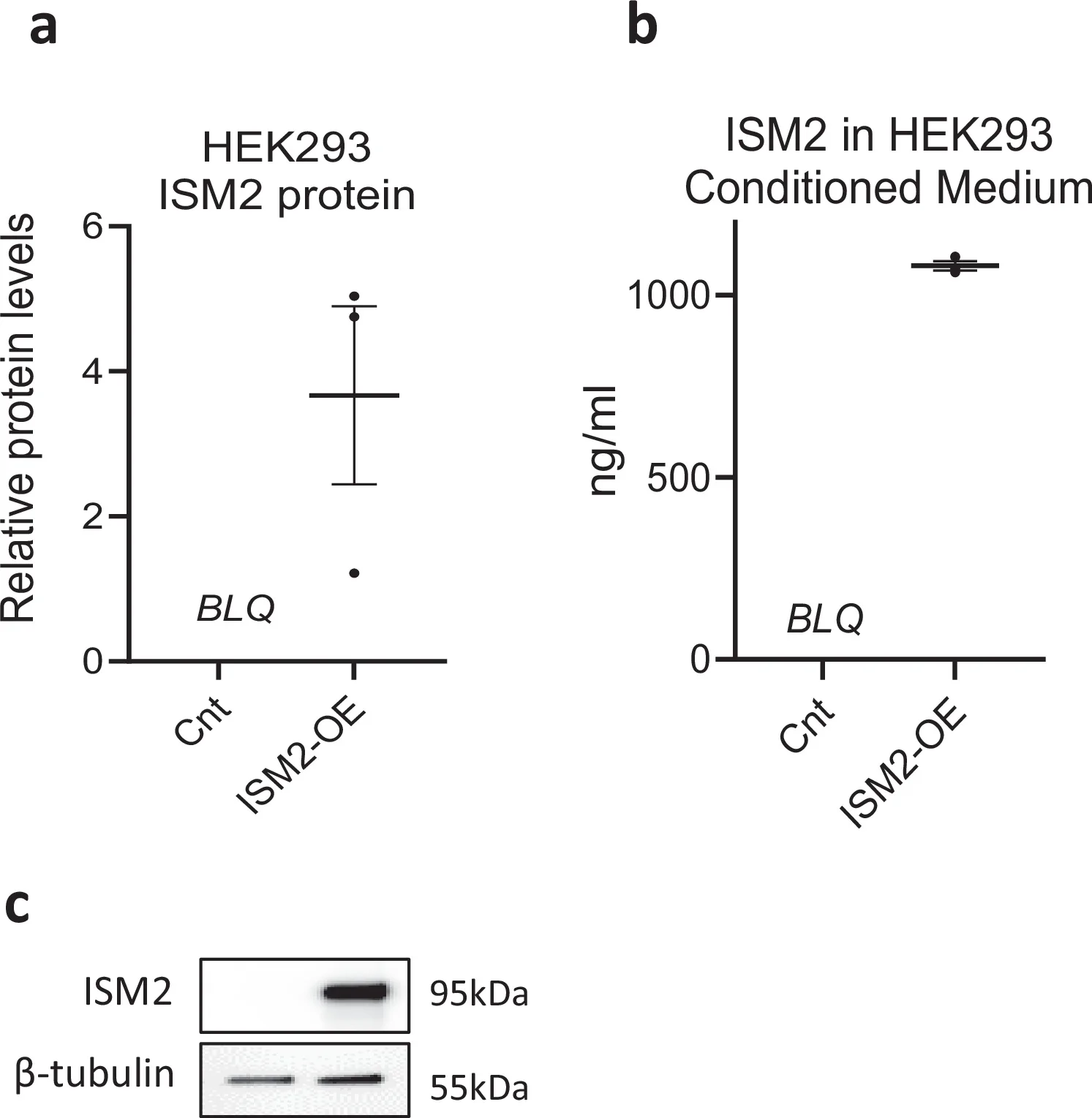
Validation of ISM2 overexpression in HEK293 cells. Validation of *ISM2* overexpression in HEK293 cells showing **a**. cellular ISM2 levels and **b**. ISM2 in conditioned media measured using Proximity Extension Assay (Olink). **c**. Immunoblot of the cell lysate as shown in a. *n* = 3, graphs show mean ± SEM. *BLQ*, below the level of quantification. Source data

In hTSC-differentiated EVTs in which *ISM2* had been knocked down, EVT morphology was rescued by the addition of recombinant ISM2 in a dose-dependent manner (Fig. 4a), with the highest doses in the physiological range. Consistent with the impairment in EVT phenotype, *ISM2* knockdown also reduced the invasive capacity of EVT into a Matrigel matrix (Fig. 4b). We then studied the effect of expressing ISM2 using lentiviral transduction in HEK293 cells (Extended Data Fig. 8a,b) that lack endogenous ISM2. In the wound healing assay, cells expressing ISM2 demonstrated enhanced migration compared with control cells (Fig. 4c). Similarly, when control HEK293 cells were exposed to the conditioned media obtained from HEK293 cells overexpressing ISM2, the unmanipulated cells demonstrated increased invasion (Fig. 4d). Collectively, these experiments demonstrated that ISM2 released from expressing cells is necessary for promoting EVT invasion and is sufficient to promote migration in a cell type that does not normally express ISM2.

## Discussion

The key findings of the current analysis are that low ISM2 was the strongest first-trimester protein signal of 2,904 targets studied in relation to PE and FGR and that the protein appeared to have a critical role in determining the invasiveness of EVT. The findings are consistent with a causal role of low levels of this protein in the early pathophysiology of PE and FGR. Several features of the analysis support a causal association. First, the association between ISM2 and the clinical outcomes was the strongest of 2,904 proteins studied. It was the only protein that was associated with all four outcomes. Moreover, it was the top predictor of three of the outcomes and the second top predictor of the fourth, and the associations were virtually identical in independent validation cohorts. Second, the direction of association was consistent with the known pathophysiology: low levels of the protein would be expected to be associated with impaired trophoblast invasion, which is thought to be causally involved in the development of both PE and FGR. Similarly, ISM2 levels were lower in cases with high-resistance patterns of uterine artery blood flow. Third, we could directly demonstrate that ISM2 was necessary for EVT invasion and sufficient to induce an invasive phenotype in an ISM2-negative cell. Fourth, the temporal nature of the association was consistent with causality. Trophoblast invasion occurs as a progressive process, starting with the decidua in the first trimester of pregnancy and extending into the myometrium and being completed in the second trimester^23^. Consistent with this, the association between low ISM2 and later disease was strongest at ~12 wkGA and was still observed at ~ 20 wkGA, but was not observed at later GAs. Indeed, at 36 wkGA, the association had reversed, with higher levels being disease-associated. This last observation is contradictory to that of the single previous publication that has analyzed ISM2 in PE in which the authors found lower levels in women who had been diagnosed with PE in late pregnancy^24^. However, this association was based on only 30 cases of PE and used a poorly characterized assay. Our data confirm the specificity of the ISM2 assay used here. ISM2 was undetectable by the Olink PEA in EVTs following shRNA-mediated knockdown (Fig. 3d). Transfection of cells that do not express ISM2 (HEK293) resulted in robust ISM2 expression that was readily detectable by the Olink assay (Extended Data Fig. 8). ISM1, which is measured by the Olink Explore panel, showed no disease association.

A limitation of the study is that the analyses are focused on northern European cohorts, and further studies should assess the association in other populations. The current study does not shed light on what causes first-trimester levels of ISM2 to be lower in women who subsequently develop PE and/or FGR, and this will be a focus for further analysis. The EVT demonstrates the highest levels of expression of ISM2. Understanding the pathways that control its secretion could shed light on the question. EVT invasion is a complex process, and there is extensive crosstalk between EVT and maternal tissues in general and uterine natural killer (uNK) cells in particular^25^. GSEA identified IFNα and IFNγ pathways both in the analysis of PE and FGR DAPs and in the transcripts that were differentially regulated by *ISM2* knockdown in EVT. Natural killer cells are a main source of IFNγ and respond profoundly to IFNα^26^. uNK cell dysfunction has been implicated in defective placentation, and cytokines produced by uNK cells (XCL1, CSF2, CSF1 and CCL5) have receptors on EVT that alter the expression of several proteins regulating EVT function. However, a study of the effect of a cocktail of these cytokines on EVT did not identify ISM2 as one of the proteins thus regulated^27^, suggesting that ISM2 may be upstream of this control system. We demonstrated that the receptors for some of these cytokines (XCR1 and CSF2RA) are reduced following ISM2 knockdown in differentiating trophoblast stem cells, so the loss or reduction of ISM2 might diminish EVT responsiveness to cytokine stimulation. Another possible explanation for low circulating ISM2 levels is that ISM2 released by the EVT is inactivated. Angiotensin II type 1 receptor and other autoantibodies have been reported to be associated with PE^28^, but there are no reports, to our knowledge, of the analysis of ISM2 autoantibodies in women who subsequently develop PE. If the mother developed neutralizing autoantibodies to ISM2, EVT invasion could be inhibited, resulting in an increased risk of later complications such as PE and FGR.

There is very little known about ISM2 other than that its expression is highly specific to the placenta and, within the placenta, to the EVT. Hence, the mechanism by which ISM2 stimulates invasion by both EVT and HEK cells remains unknown. ISM2 shares 46% amino acid sequence homology with ISM1 (ref. ^29^). ISM1 promotes cell migration and renal branching by binding to integrins^30^. Given the close homology with ISM1, particularly in the domains implicated in integrin binding, ISM2 may also promote cell migration by interacting with integrins, and this would be consistent with a large body of evidence that indicates a key role for integrins in EVT invasion^31^. For example, cytotrophoblast differentiation into invasive EVTs is accompanied by spatial and temporal changes in the integrin repertoire in vivo, a process referred to as ‘integrin switching’. Consistent with this, upregulation of integrins *ITGA5* and *ITGA1* with hTSCs differentiating into EVTs was prevented by *ISM2* knockdown.

The current study has potential implications for therapeutic development. Therapeutic interventions to enhance EVT secretion of ISM2 would be potentially useful in women at high risk of PE or FGR in the first half of pregnancy. Given that ISM2 can stimulate cell migration in other cell types, systemic administration of the protein may be associated with off-target effects. However, recent studies have reported using placenta-trophic lipid nanoparticles to deliver mRNA to the placenta^32^. Moreover, EVT has unique surface proteins (for example, HLA-G) that may facilitate targeting lipid nanoparticles to this cell type. Conversely, neutralizing humanized monoclonal antibodies could be useful in clinical situations in which the inhibition of trophoblast invasion is desirable. Examples of this include the management of ectopic pregnancy and cesarean scar pregnancy and, potentially, post-coital or other contraception. Such a treatment might be expected to have a low potential for off-target effects given the specificity of ISM2 for EVT^33^.

In conclusion, we show that low ISM2 in the first trimester of pregnancy had the strongest association with the subsequent experience of PE and/or FGR among 2,904 proteins studied in maternal serum. A causal association between low levels of this protein and disease is supported by the facts that the protein is necessary for EVT invasion and that failure of trophoblast invasion of the uterus is thought to be a crucial element of the etiology of both conditions.

## Methods

### Patient and sample selection

#### Cohort studies

The current study used samples from three cohorts. The only exclusion criterion was that the pregnancy ended in stillbirth. The primary source of data and biological samples was the POPS. The study protocol has been published^34^, and the data and biological samples collected from the study have been summarized elsewhere^8,9^. The study was conducted in the Rosie Hospital, Cambridge, UK, between 2008 and 2013. Ethical approval was obtained from the Cambridgeshire 2 Research Ethics Committee (reference number 07/H0308/163). All study participants gave written informed consent. In brief, 4,512 nulliparous women with a singleton pregnancy were recruited and 4,212 were followed through to delivery. Participation involved obtaining blood at the time of recruitment (typically ~12 wkGA) and attending for visits at ~20 wkGA, ~28 wkGA and ~36 wkGA. At each of these visits, an obstetric ultrasound scan was performed and blood was obtained, which was processed into serum and plasma. The definition of maternal characteristics has been previously described^8,9^. The mean uterine artery pulsatility index at ~20 wkGA was log-transformed and turned into an exact GA-adjusted *z* score^8^. Five first-trimester biomarkers (PAPPA, PlGF, sFLT1, hCG, and AFP) were measured in maternal serum samples using Roche Eclesys assays on the electrochemiluminescence immunoassay platform, Cobas e411 (Roche Diagnostics)^35^. For the present study, the biomarkers were log_2_-transformed, turned into exact GA-adjusted *z* scores referent to the random comparison group, and winsorized at −5 and +5. After the delivery, samples of the placenta and membranes were obtained, and the outcome of the pregnancy was ascertained by assessment of the patient’s clinical record, the neonatal record and associated laboratory information. The POPS2 cohort was used for validation of findings from POPS. This study has been described elsewhere (https://doi.org/10.1186/ISRCTN12181427) but is still ongoing, having started in 2020 and scheduled to complete recruitment in 2026. Ethical approval was obtained from East of England – Essex Research Ethics Committee (reference number 19/EE/0331). All study participants gave written informed consent. The model for data and sample collection is identical to the POPS cohort, but POPS2 differs in that the study also includes a nested randomized controlled trial. Women are screened at 36 wkGA to assess their risk of PE and FGR, and high-risk women are randomized to either have the result revealed or masked. When the result is revealed, the risks are discussed and the participant is offered enhanced monitoring and/or earlier delivery. When the result is masked, their high-risk status is not revealed and they have routine care. For the purposes of the present analysis, we excluded non-cases who screened high risk and had the screening result revealed, and we used data and samples selected from the first 2,595 participants who had completed the study out of a total target recruitment of 5,500. Additionally, we used data and samples from 432 participants in the IMPACT study^16^, a Swedish multicenter prospective cohort in which unselected pregnant women with a Swedish personal identity number were recruited between 11 wkGA and 14 wkGA. Medical history and demographic data were obtained at enrollment, along with maternal blood and the results of physical examination. Research data were linked to routinely collected data held in the Swedish Pregnancy Register, and this included ascertainment of pregnancy outcome. The IMPACT study has been granted national ethical approval by the Swedish Ethical Review Authority (Uppsala 2018-231) and national biobank approval at Uppsala Biobank (18237 2 2018 231). None of the cohorts paid participants other than reimbursement of travel expenses.

#### Case–cohort analysis and case definition

The experimental design of the present analysis was a case–cohort study, as previously described^10,11^. In the POPS and POPS2 cohorts, case status was defined as women experiencing PE only, FGR only or both conditions. We also studied a composite of any of the three outcomes. For the purposes of the present analysis, PE was defined as per the 2013 American College of Obstetricians & Gynecologists (ACOG) Guideline^36^, and cases were confined to those that either resulted in preterm delivery (<37 wkGA) or those in which there were severe features, as defined by the ACOG guideline, in women with no previous history of essential hypertension or renal disease (that is, de novo PE, excluding superimposed disease). FGR was defined as a birth weight <3rd percentile or birth weight <10th percentile combined with a complication, specifically, either preterm birth or PE. In POPS, birth weight percentile was defined by a customized standard (including fetal sex and maternal characteristics) based on the Hadlock fetal growth^37^. In POPS2, birth weight percentile at term was defined by a UK 1990 birth weight reference standard^38^, and birth weight percentile at preterm was defined by the Hadlock fetal growth^39^ customized for fetal sex only (the fetal sex adjustment was based on the differences between males and females in POPS). In the POPS and POPS2 cohorts, we identified a random sample of the cohort with a known sampling fraction. In a given analysis, women in the random sample who had the outcome of interest were re-labeled as cases, and all cases were then compared with the remaining women in the random sample who did not experience PE or FGR. In the IMPACT study, the outcome was defined as PE complicated with FGR and/or preterm birth, and these cases were compared with the remaining women in the random sample who did not experience any type of PE or FGR. The IMPACT study defined PE using ICD-10 codes O11, O14 and O15, and FGR as birth weight ≤10th percentile for GA and fetal sex^40^, using data held in the Swedish Pregnancy Register.

#### Proteomics

The discovery analysis used stored serum samples from the POPS cohort that were analyzed by Olink (Uppsala, Sweden). Olink performed the Explore assay (v3.6) that estimates the relative concentration of 2,944 proteins in a sample — a total of 2,904 proteins passed the quality control, which we used in the main analysis. Protein levels are quantified relatively (as opposed to absolute concentrations), expressed as NPX units, an arbitrary number on a log_2_ scale. Olink used the PEA method that is described in detail elsewhere^41^. In brief, pairs of antibodies for a given protein are linked to complementary oligonucleotides with a sequence that is specific for the given protein. The antibodies are incubated with the sample, and, in the presence of the protein, the complementary oligonucleotides are brought into close proximity and hybridize. The gaps in the duplex are filled in by DNA polymerase, and the concentration of protein in the sample is quantified by sequencing or PCR analysis of the extended oligonucleotides. In the POPS cohort, the Explore assay was performed on all available serum samples for all participants who fulfilled the case definition and for women in the randomly selected comparison group. Validation also used the Olink Explore panel, but this was a slightly later iteration of the assay (v3.10), measuring 2,943 proteins in which 2,859 of them passed the quality control. Overall, there were 2,840 unique proteins shared in both panels that we used in validation analysis. The current analysis focuses on samples obtained at ~12 wkGA. However, ISM2 levels were also measured at ~20 wkGA, ~28 wkGA and ~36 wkGA using the same method. Finally, we used a custom-built Focus panel (Olink) that uses the same antibodies as the Explore panels^42^. The custom Focus panel was manufactured by Olink and included assays for ISM2 and PlGF. This assay has a qPCR endpoint and was run according to the manufacturer’s instructions on the Olink Signature Q100 microfluidic/qPCR instrument. This assay included standards with known concentrations to allow construction of a standard curve and quantitative analysis of protein levels.

**Extended Data Fig. 9:**
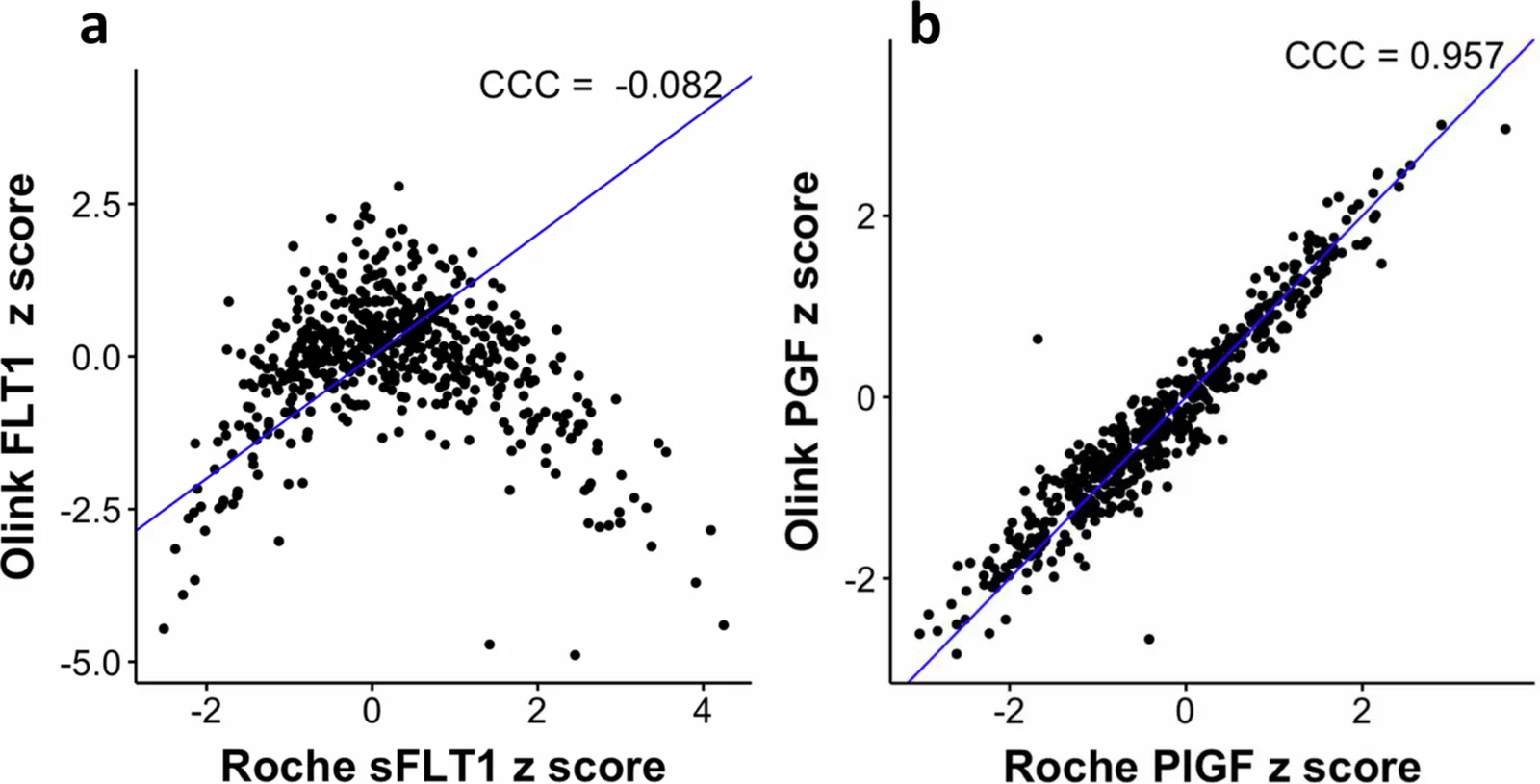
Measurement of sFLT1 and PlGF by the Olink Explore assay and the Roche Cobas 411e in the POPS cohort. Plots of Roche cobas measurement and Olink Explore measurement of **a**. sFLT1, **b**. PlGF at 36wkGA (n = 554), where CCC denotes Concordance Correlation Coefficient. In the Olink analyses, sFLT1 and PlGF are labelled as FLT1 and PGF, respectively. Source data

In previous studies, we have analyzed maternal sFLT1 and PlGF in serial samples from the POPS cohort^43,44^. The Olink Explore assay measures these proteins, but the level of sFLT1 is greatly elevated in pregnancy and outside the linear range of the Olink assay (Extended Data Fig. 9a). We therefore used our previous measurements of sFLT1 made using the Roche Cobas 411e analysis platform. The Roche measurement was expressed in picograms per milliliter, and this was log_2_-transformed and converted to a GA-adjusted *z* score, to make it comparable with the Olink data. The pregnancy PlGF levels are within the Olink assay’s dynamic range and showed a high level of agreement with the Roche assay (Extended Data Fig. 9b), so these were used without modification. In the Olink analyses, sFLT1 and PlGF were labeled as FLT1 and PGF, respectively.

### Statistical analysis

#### Processing of data and management of outliers

The gestational windows of blood sampling in the POPS and POPS2 were ~12 wkGA, ~20 wkGA, ~28 wkGA and ~36 wkGA. However, at each time point, there was a degree of variation in the exact GA. We calculated a GA-adjusted *z* score for each protein concentration within each time window, referent to the whole of the random sub-cohort. All subsequent analyses used the GA-adjusted *z* score. We identified individual extreme outliers in a Principal Component Analysis performed using GA-adjusted *z* scores for all the samples (cases and controls) from a given cohort collected at one of the four time points. Extreme outliers were identified by a visual inspection in a plot of PC1 and PC2 and were dropped in subsequent analyses. GA-adjusted *z* scores were recalculated if any of the excluded extreme outliers had been in the random comparison group. To limit the influence of extreme outliers, all *z* scores were winsorized^45^ at −5 and +5. A similar procedure was applied to the IMPACT study samples collected between 11 wkGA and 14 wkGA.

#### Univariate analysis

We performed univariate analysis for the disease association between each protein and each case type by both a simple *t*-test and by logistic regression. We defined DAPs as proteins that were statistically significantly associated with case status by either *t*-test or logistic regression using the Benjamini–Hochberg method of false discovery rate correction (5%). The distribution of *p* values from the *t*-test was plotted as a frequency histogram, and we used the Kolmogorov–Smirnov test to determine whether there was a statistically significant excess of low *p* values. We also created volcano plots for each of the conditions at each of the gestational time points, with the mean difference in NPX *z* score between the cases and controls on the *x* axis and −log_10_(*p*) on the *y* axis, where the *p* value was calculated using a *t*-test, after correcting for false discovery (5%) using the Benjamini–Hochberg method.

#### Development of multivariable predictive models

We performed a literature review and identified relevant statistical and machine-learning methods that we used to build multivariable predictive models. These included regularized logistic regression, random forest, *k*-nearest neighbors, multivariate adaptive regression splines, support vector machines, Gaussian process classification models, discriminant analyses, extreme gradient boosting (XGBoost), and various neural network methods, which were compared with ISM2 as a sole predictor (Supplementary Table 9). The predictive models were trained to predict the binary composite outcome in the POPS dataset, and they were externally evaluated in the independent POPS2 dataset. We used the glmnet (v4.1.2) R package to fit the regularized logistic regression models. For the remaining methods, we used the caret R package (v6.0.94) to tune and optimize parameters and hyperparameters using tenfold cross-validation, with a preprocessing step to remove correlated predictors (a correlation cutoff of 0.75) and near-zero variance predictors. We identified the AUC as the indicator of model discrimination for selecting the best performing model, which was calculated together with CIs using the pROC (v1.18.5) R package.

#### External evaluation of protein associations and predictive models

We performed GA normalization and identified outliers in the POPS2 cohort using the same methods as used in the POPS cohort. For validation of univariate associations, we created *x*/*y* plots for all of the DAPs in POPS, with the univariate beta coefficients from logistic regression in POPS on the *x* axis and the univariate beta coefficients from logistic regression in POPS2 on the *y* axis. To evaluate each of the multivariable predictive models, we estimated the predicted probability of disease for each woman in POPS2 based on the model developed in POPS. We then calculated the AUC and 95% CIs in POPS2 using the predicted probabilities. We compared AUCs for the multivariable model versus ISM2 as a sole predictor using the DeLong test^46^. Finally, we compared the predictive performance of ISM2 and PlGF as sole predictors of complicated PE in the IMPACT study, using AUCs and the DeLong test.

#### Analysis of effects of ISM2 in cell culture

##### Cell culture

Cells from the hTSC line CT27, previously derived from first-trimester human placentas, were a kind gift from Dr Hiroaki Okae (Institute of Molecular Embryology and Genetics, Kumamoto University). These hTSCs were cultured in their stem state or differentiated into STB or EVTs as previously described^5,47^. For EVT organoid formation, hTSCs were grown to ~80% confluence, cells were dissociated with TrypLE to obtain single cells, and 5,000 cells were suspended in 40-µl Matrigel droplets in 24-well plates. After 5 min of incubation, the plates were turned upside down and placed in a 37 °C incubator for 15 min to allow the Matrigel droplets to polymerize into dome shapes. The plates were then removed from the incubator and turned over, and 500 µl of trophoblast organoid medium^6^ was added to them, after which they were returned to the 37 °C, 5% CO_2_ incubator. The trophoblast organoid medium was changed every other day for 6 days, after which it was replaced with EVT medium-1 (Advanced DMEM/F12, 0.3% bovine serum albumin (BSA), 4% knockout serum replacement, 1× ITS-X, 0.1 mM 2-mercaptoethanol, Primocin (100 µg ml^−1^), 7.5 μM A83-01, 2.5 µM Y-27632, and 100 ng ml^−1^ NRG1). EVT medium-1 was replaced every other day for a total of 5 days, after which the organoids were replaced with EVT medium-2 (identical to EVT medium-1 but with the omission of NRG1). After 6 days in culture with media changes every other day, EVT organoids were imaged while they were still in the Matrigel or fixed in 4% paraformaldehyde for immunocytochemistry. Trophoblast organoids were grown in their standard conformation (apical-in) with the STB facing the inside core and TSCs facing the Matrigel on the outside to allow EVTs to differentiate and migrate from the TSCs^6,27,48^.

HEK293 cells (a kind gift from Dr Roberta Azzarelli, School of Pharmacy, University College London) were grown in DMEM (high glucose) with 10% fetal bovine serum and 1× penicillin–streptomycin–glutamine. Culture media was changed every other day and cells were passaged at ~80% confluency.

##### *ISM2* knockdown and transduction

*ISM2* knockdown was performed in hTSCs by lentiviral transduction with *ISM2*-shRNA or Scr-shRNA (controls) vectors carrying EGFP for selection. Scr-shRNA (pLV[shRNA]-EGFP-U6 > Scramble-shRNA) and *ISM2*-shRNA (pLV[shRNA]-EGFP-U6 > ISM2-shRNA) lentiviral particles were obtained from VectorBuilder (IDs VB010000-0001mty and VB240726-1053qhc, respectively). The hTSCs were plated at 100,000 cells per well in six-well plates and transduced at a multiplicity of infection of 5. After 24 h in 37 °C, 5% CO_2_ incubator, media was replaced and cultured for a further 48 h before sorting for EGFP fluorescence by fluorescence activated cell sorting (FACS) at the Cambridge BRC Phenotyping Hub. Lentiviral transduction of ISM2 in HEK293 cells was performed using ISM2 vector (pLV[Exp]-EGFP:T2A:Puro-EF1A > hISM2; ID: VB900022-6681dfw) or an empty vector control (pLV[Exp]-EF1A > EGFP: VB900088-2243bzq) at a multiplicity of infection of 5.

##### Matrigel invasion assay

Trophoblast invasion into Matrigel was performed using the chamber-based technique as previously described^49^. Briefly, secure-seal hybridization chamber gaskets (Thermo Fisher Scientific, S24732) were UV sterilized and attached to 12-well plates. Matrigel was combined with EVT medium (at a 2:1 ratio), and 17 µl of this mixture was loaded through one chamber port. The Matrigel mixture was polymerized by incubating the chambers for 10 min at 37 °C, and 60,000 hTSCs (either Scr-shRNA or ISM2-shRNA transduced) suspended in 25 µl of EVT media were added through the other chamber port. The chambers were incubated tilted in a 45° angle for 25 min to promote cell attachment along the Matrigel interface. The cultures were then overlaid with EVT medium and maintained at 37 °C and 5% CO_2_. The media was then replaced with the different EVT media according to the differentiation protocol^5,47^. Cultures were maintained for 12 days when the images were acquired using the EVOS M5000 microscope (Invitrogen). Image quantification and analysis were performed using Fiji (ImageJ) software.

##### Wound healing (scratch) assay

HEK293 cells were plated at 50,000 cells per well of six-well plates. After 24 h, when the cells were near-confluent (>80% confluency), a region of the same length and width was scratched. The culture media was immediately replaced with either fresh media (for knockdown and overexpression experiments) or conditioned media from ISM2-OE or Cnt cells diluted 1:1 with fresh media (for conditioned media experiments). Images were captured with a microscope under ×10 magnification at 0 h and 24 h after the scratch was performed. The percentage of scratch closure was measured by dividing the uncovered area at 24 h by the initial area at 0 h. Quantification of scratch closure was performed using wound healing size plugin in ImageJ software (version 1.54p).

##### Immunocytochemistry

The Matrigel droplets containing EVT organoids were digested using Cell Recovery Solution (Corning #354253) and washed gently in PBS buffer. The organoids were fixed in 4% paraformaldehyde on ice for 30 mins, washed in PBS buffer with 0.1% tween (PBS-T), and blocked in PBS-T with 1% BSA. Organoids were incubated with rabbit anti-ISM2 antibody (Proteintech #21540-1-AP) and mouse anti-HLA-G antibody (Bio-Rad #MCA2044, clone MEM-G/9) at 1:100 dilution (for both antibodies) in PBS-T with 1% BSA at 4 °C overnight. After several washes, fluorescent secondary antibodies (Alexa Fluor-488 anti-rabbit IgG #A-21441 and Alexa Fluor-568 anti-mouse IgG #A11004; Thermo Fisher Scientific) were added at 1:400 dilution for 2 h at room temperature. Organoids were then washed before adding optical clearing solution (60% vol/vol glycerol and 2.5 M fructose) for 20 min. Organoids were mounted in Prolong Glass Antifade Mountant with NucBlue stain (Thermo Fisher Scientific #P36981). Imaging was performed on a Nikon Eclipse Ti2 fluorescent microscope.

##### RNA isolation, cDNA synthesis and quantitative PCR

Total RNA was isolated and genomic DNA removed using the RNeasy Plus mini kit (Qiagen #74134), and cDNA synthesis performed using the high-capacity cDNA reverse transcription kit (Thermo Fisher Scientific #4368814). Quantitative PCR was performed using TaqMan multiplex master mix (Thermo Fisher Scientific #4461881) with pre-designed TaqMan assays using the FAM reporter dye (*ISM2*, Hs01071575; *ERVW-1*, Hs00205893; *CYP19A1*, Hs00903411; *GDF15*, Hs00171132). Target gene expression was normalized to the geometric mean of reference genes *TBP* and *CDC34* (Hs00427620 and Hs00362082, respectively) labeled with the VIC reporter dye.

##### RNA sequencing and data processing

RNA quality was assessed using the RNA screen tape on the TapeStation (Agilent Technologies), and all samples had an RNA integrity number of 10 and were thus suitable for RNA sequencing. We used 100 ng RNA per sample to generate barcoded sequencing libraries with the Illumina Stranded mRNA prep ligation kit (#20040534) and the Illumina index kit A (#20091655). The quality of the cDNA libraries was validated using the DNA screen tape. Indexed libraries were pooled and sequenced with a 50-bp paired-end protocol on a NovaSeqX platform (Illumina) in the Cancer Research UK Cambridge Institute Genomics Core Facility, and the quality of reads was assessed using MultiQC (v1.19). Transcript quantification was performed using Salmon (v1.10) in selective-alignment mode with a decoy-aware transcriptome indexed using the Ensembl 88 version of transcript annotations. The transcript-level read count matrices were imported using tximeta (v1.20.3) Bioconductor package and merged at the gene level. We considered only genes found in ≥50% of samples having at least ten reads and discarded genes detected as dispersion outliers by DESeq2 (v1.42.1). We identified outlier samples that were not clustered with the same cell types by a visual inspection in a plot of Principal Component Analysis; they were dropped in subsequent analyses. A total of 36 samples (*ISM2*-shRNA and Scr-shRNA paired samples in *n* = 8 in each of hTSC, STB, EVT mid, and *n* = 12 in EVT terminal) were used to find DEGs in each cell type in paired design (that is, ~Pair+Treatment) of DESeq2. The *p* values were calculated from the null hypothesis that the fold changes were less than or equal to 20% (that is, lfcThreshold = log_2_(1.2)). For edgeR (v4.0.16) analysis, we used the ‘makeDGEList’ function of tximeta Bioconductor package to convert the data object of the 36 samples as mentioned above. The gene-level count matrix was normalized by using the ‘calcNormFactors’ function of edgeR with the trimmed mean of *M* values method, and a quasi-likelihood negative binomial generalized log-linear model (that is, glmQLFit) was applied. For a statistical test, we used the ‘glmTreat’ function of edgeR with at least 20% fold change (that is, lfc = log_2_(1.2)). For GSEA, we used clusterProfiler (v4.10.1) based on the 17,802 genes ranked in their descending order using the values from the following formula: $\mathrm{sign}({\mathrm{log}}_{2}\mathrm{FC})\times -{\mathrm{log}}_{10}(p)$, where log_2_FC and *p* value were from edgeR. The hallmark (collection ‘H’) and cell-type signature (collection ‘C8’) gene sets were downloaded using ‘msigdbr’ (v10.0.1) Bioconductor R package, and gene sets with a minimum size of 20 genes were considered in GSEA.

##### Western blotting

Cells were harvested in radioimmunoprecipitation buffer (50 mM Tris HCl, pH 7.4; 150 mM NaCl; 0.1% SDS; 0.5% Na-deoxycholate and 1% Triton X-100) containing protease and phosphatase inhibitor cocktail (Fisher Scientific #15624189). Protein lysates were denatured under reducing conditions, separated on 4%–15% TGX gels (Bio-Rad #4561086), and transferred to polyvinylidene difluoride membranes. Amido Black staining was performed and membranes imaged for assessment of equal protein loading before blocking in 10% BSA in Tris-buffered saline with 0.1% Tween (TBS-T). Membranes were then incubated in rabbit anti-ISM2 (Proteintech #21540-1-AP) at 1:1,000 dilution in TBS-T with 2% BSA at 4 °C overnight or with mouse anti-β-tubulin (clone D3U1W, Cell Signaling Technology #86298) at 1:2,000. After several washes in TBS-T, membranes were incubated with peroxidase-conjugated secondary antibodies (anti-mouse or anti-rabbit; Cell Signaling Technology #7076 and #7074, respectively) at 1:2,000 dilution in TBS-T with 2% BSA for 2 h, washed and visualized by enhanced chemiluminescence using Clarity Western ECL substrate on the iBright FL1500 imager. Target protein levels were normalized against β-tubulin.

### Reporting summary

Further information on research design is available in the Nature Portfolio Reporting Summary linked to this article.

## Online content

Any methods, additional references, Nature Portfolio reporting summaries, source data, extended data, supplementary information, acknowledgements, peer review information; details of author contributions and competing interests; and statements of data and code availability are available at https://doi.org/10.1038/s41591-026-04573-6.

## Supplementary information

Reporting Summary

Peer Review file

Supplementary Tables 1–14 Supplementary tables, including an index.

## Source data

Source Data Fig. 1 Statistical source data.

Source Data Fig. 2 Statistical source data.

Source Data Fig. 3 Statistical source data and uncropped blots.

Source Data Fig. 4 Statistical source data.

Source Data Extended Data Fig./Table 1 Source data for Extended Data Fig. 1.

Source Data Extended Data Fig./Table 2 Source data for Extended Data Fig. 2.

Source Data Extended Data Fig./Table 3 Source data for Extended Data Fig. 3.

Source Data Extended Data Fig./Table 4 Source data for Extended Data Fig. 4.

Source Data Extended Data Fig./Table 5 Source data for Extended Data Fig. 5.

Source Data Extended Data Fig./Table 6 Source data for Extended Data Fig. 6.

Source Data Extended Data Fig./Table 7 Source data for Extended Data Fig. 7.

Source Data Extended Data Fig./Table 8 Source data for Extended Data Fig. 8.

Source Data Extended Data Fig./Table 9 Source data for Extended Data Fig. 9.

## Data Availability

The RNA-seq datasets generated in this study are available in the European Nucleotide Archive (ENA, www.ebi.ac.uk/ena) under accession numbers PRJEB89714 and PRJEB110901. Source data for Figs. 1–4 and Extended Data Figs. 1–9 are available online. Publication of individual patient data and clinical characteristics is precluded by the ethics approval. Source data are provided with this paper.

## Extended data

are available for this paper at https://doi.org/10.1038/s41591-026-04573-6.

## Code availability

All custom code used for the current study is publicly available: https://github.com/ObsGynaeCam/ism2-2025.
