# Supplementary material for: Isthmin-2 is a first-trimester predictor of preeclampsia and fetal growth restriction
Source: Nat Med. 2026 Sep 1;32(9):3351–8. doi: 10.1038/s41591-026-04573-6 (PMC13577898; doi:10.1038/s41591-026-04573-6)
Supplement: Supplementary file 1 [file 41591_2026_4573_MOESM1_ESM.pdf]

Corresponding author(s): D. Stephen Charnock-Jones and Gordon C.S. Smith

Last updated by author(s): Jun 10, 2026

Reporting Summary

Nature Portfolio wishes to improve the reproducibility of the work that we publish. This form provides structure for consistency and transparency in reporting. For further information on Nature Portfolio policies, see our Editorial Policies and the Editorial Policy Checklist.

Statistics

For all statistical analyses, confirm that the following items are present in the figure legend, table legend, main text, or Methods section.

n/a	Confirmed	
☐	☒	The exact sample size (*n*) for each experimental group/condition, given as a discrete number and unit of measurement
☐	☒	A statement on whether measurements were taken from distinct samples or whether the same sample was measured repeatedly
☐	☒	The statistical test(s) used AND whether they are one- or two-sided<br>*Only common tests should be described solely by name; describe more complex techniques in the Methods section.*
☐	☒	A description of all covariates tested
☐	☒	A description of any assumptions or corrections, such as tests of normality and adjustment for multiple comparisons
☐	☒	A full description of the statistical parameters including central tendency (e.g. means) or other basic estimates (e.g. regression coefficient) AND variation (e.g. standard deviation) or associated estimates of uncertainty (e.g. confidence intervals)
☐	☒	For null hypothesis testing, the test statistic (e.g. *F*, *t*, *r*) with confidence intervals, effect sizes, degrees of freedom and *P* value noted<br>*Give P values as exact values whenever suitable.*
☒	☐	For Bayesian analysis, information on the choice of priors and Markov chain Monte Carlo settings
☒	☐	For hierarchical and complex designs, identification of the appropriate level for tests and full reporting of outcomes
☐	☒	Estimates of effect sizes (e.g. Cohen's *d*, Pearson's *r*), indicating how they were calculated

Our web collection on statistics for biologists contains articles on many of the points above.

Software and code

Policy information about availability of computer code

Data collection	The software used to collect data were the standard NovaSeq (Illumina) sequencing machine software, the quantitative PCR machine software (QuantStudio 6 Flex system, ThermoFisher Scientific), and ImageJ software (version 1.54p).
Data analysis	MultiQC (v1.19), Salmon (v1.10), Ensembl 88 version of transcript annotations, tximeta (v1.20.3), DESeq2 (v1.42.1), edgeR (v4.0.16), clusterProfiler (v4.10.1), pheatmap (v1.0.12), msigdbr (v10.0.1), glmnet (v4.1.2), caret (v6.0.94), pROC (v1.18.5), mclust (v6.1.1), randomForest (v4.7.1.1), earth (v5.3.2), MASS (v7.3.58.2), kernlab (v0.9.32), xgboost (v1.7.8.1), RSNNS (v0.4.17), nnet (v7.3.19), stats(4.2.3), R (v4.2.3), Stata (v18.0)

For manuscripts utilizing custom algorithms or software that are central to the research but not yet described in published literature, software must be made available to editors and reviewers. We strongly encourage code deposition in a community repository (e.g. GitHub). See the Nature Portfolio guidelines for submitting code & software for further information.

Data

Policy information about availability of data

All manuscripts must include a data availability statement. This statement should provide the following information, where applicable:

- Accession codes, unique identifiers, or web links for publicly available datasets
- A description of any restrictions on data availability
- For clinical datasets or third party data, please ensure that the statement adheres to our policy

The RNA-Seq dataset generated in this study are available in the European Nucleotide Archive (ENA www.ebi.ac.uk/ena) under the accession numbers PRJEB89714 and PRJEB110901. Source data for Figs. 1-4 and Supplementary Figs. 1-9 are available online. Individual patient data and clinical characteristics cannot be provided as this may compromise patient confidentiality and is precluded by the ethics approval.

Research involving human participants, their data, or biological material

Policy information about studies with human participants or human data. See also policy information about sex, gender (identity/presentation), and sexual orientation and race, ethnicity and racism.

Reporting on sex and gender	Pregnant women were recruited to the POPS, POS2 and IMPACT cohorts. Birth weight percentiles were customized for fetal sex.
Reporting on race, ethnicity, or other socially relevant groupings	No analyses were performed which used ancestry as a variable.
Population characteristics	The POP study population was an unselected cohort of nulliparous women with a singleton pregnancy who delivered in Cambridge, UK. The cohort has been described in detail in our previous papers (Sovio et al, Lancet 2015;386(10008):2089-2097; Gaccioli et al, Placenta 2017;59(Supp1):S17-S25; Gong et al, JCI Insight 2018;3(13):e120723); Sovio et al, Nature Medicine 2020;26:348-353. POPS2 is a similar prospective cohort and collection is on-going. It has been described here https://doi.org/10.1186/ISRCTN12181427.<br>The IMPACT consortium is a prospective cohort and has been described in detail: Bergman, L. et al. Study for Improving Maternal Pregnancy And Child ouTcomes (IMPACT): a study protocol for a Swedish prospective multicentre cohort study. BMJ Open 10, e033851 (2020).
Recruitment	See above
Ethics oversight	For the POPS cohort Ethical approval was obtained from the Cambridgeshire 2 Research Ethics Committee. For POPS2 Ethical approval was obtained from the East of England - Essex Research Ethics Committee.<br>The IMPACT study has been granted national ethical approval by the Swedish Ethical Review Authority (Uppsala 2018-231) and national biobank approval at Uppsala Biobank (18237 2 2018 231).

Note that full information on the approval of the study protocol must also be provided in the manuscript.

Field-specific reporting

Please select the one below that is the best fit for your research. If you are not sure, read the appropriate sections before making your selection.

☒ Life sciences ☐ Behavioural & social sciences ☐ Ecological, evolutionary & environmental sciences

For a reference copy of the document with all sections, see nature.com/documents/nr-reporting-summary-flat.pdf

Life sciences study design

All studies must disclose on these points even when the disclosure is negative.

Sample size	Sample size was determined by the availability of cases and experiment costs in the Pregnancy Outcome Prediction (POP) study, and a nested case cohort study design was used. We chose the most severe cases (235 women experiencing preeclampsia, fetal growth restriction or both conditions) and 264 controls from the random comparison group of 325 women. Of these, 223 cases and 242 controls had maternal serum proteomics data available at 12 weeks of gestation. The nested case cohort study has been previously described (Sovio et al, Nature Medicine 2020;26:348-353).
Data exclusions	Exclusion of 300 women recruited to the POP study who withdrew from the study or were lost to follow-up has been described in detail in our previous paper (Gaccioli et al, Placenta 2017;59(Supp1):S17-S25, Fig. 1B). Of the 4212 women who completed the study, we first excluded miscarriages and intrauterine fetal deaths (n=29) and women who did not have any blood samples for analysis (n=6), leaving 4177 women eligible for the case cohort selection. 3712 were excluded from the present analysis of 12-week proteomics by design. The main analysis was performed on 223 cases and 242 controls who all delivered a liveborn baby.
Replication	Replication was performed in an external validation sample from the POPS2 Cohort. Predictive models were developed using samples from

Replication	POPS and evaluated in the POPS2 samples (78 cases and 85 controls).<br>Replication in the IMPACT cohort was performed using 144 cases and 256 controls
Randomization	Participants were allocated into groups based on pregnancy outcome. Outcome data were ascertained by review of each woman's paper case record by research midwives and by record linkage to clinical electronic databases of ultrasonography (Astraia, Munchen, Germany) and delivery (Protos, iSoft, Banbury, UK).
Blinding	Sample collection was blinded during the POPS and POPS2. Researchers were blinded to the pregnancy outcome when performing all the the downstream experiments (discovery and replication). Data were unblinded at the statistical analysis stage.

Reporting for specific materials, systems and methods

We require information from authors about some types of materials, experimental systems and methods used in many studies. Here, indicate whether each material, system or method listed is relevant to your study. If you are not sure if a list item applies to your research, read the appropriate section before selecting a response.

Materials & experimental systems

n/a	Involved in the study
☐	☒ Antibodies
☐	☒ Eukaryotic cell lines
☒	☐ Palaeontology and archaeology
☒	☐ Animals and other organisms
☒	☐ Clinical data
☒	☐ Dual use research of concern
☒	☐ Plants

Methods

n/a	Involved in the study
☒	☐ ChIP-seq
☒	☐ Flow cytometry
☒	☐ MRI-based neuroimaging

Antibodies

Antibodies used	Rabbit anti-ISM2 Proteintech #21540-1-AP; Mouse anti-beta Tubulin (D3U1W), Cell Signaling Technology #86298. Secondary antibodies: anti-mouse Cell Signaling Technology #7076; anti-rabbit; Cell Signaling Technology #7074.
Validation	The antibodies from Cell Signaling Technology have been cited >200 times (beta-tubulin) and >10,000 times (secondaries). The anti-ISM2 proximity extension assay and antibody used here was validated by knockdown and over expression (Fig 3 and Suppl Fig 8).

Eukaryotic cell lines

Policy information about cell lines and Sex and Gender in Research

Cell line source(s)	Trophoblast stem cells (CT27) derived from human first trimester placenta was a gift from Dr Hiroaki Okae, Kumamoto University (formerly at Tohoku University when the lines were derived) The CT27 line used in this study was previously published by Okae et al 2018 (PMID: 29249463). These human trophoblast stem cell line can now be obtained commercially from Riken Bioresource Centre (https://cellbank.brc.riken.jp/cell_bank/CellInfo/?cellNo=RCB4936&lang=En)<br>HEK293 cells were originally purchased from American Tissue Culture Collection (ATCC) and provided as a gift from Dr Roberta Azzarelli, University College of London. Information about the cell line can be found https://www.atcc.org/products/crl-1573.
Authentication	Authentication of the hTSC line is reported in Okae et al. 2018 (PMID: 29249463), and https://knowledge.brc.riken.jp/xsearch/catalog/list?__lang__=en&query=human+trophoblast+stem+cells.<br>Authentication information of HEK293 can be obtained from the ATCC website using the links provided above.
Mycoplasma contamination	All lines tested negative for mycoplasma contamination using LookOut Mycoplasma PCR detection kit (MP0035, Sigma).
Commonly misidentified lines<br>(See ICLAC register)	None

Plants

Seed stocks	*Report on the source of all seed stocks or other plant material used. If applicable, state the seed stock centre and catalogue number. If plant specimens were collected from the field, describe the collection location, date and sampling procedures.*
Novel plant genotypes	*Describe the methods by which all novel plant genotypes were produced. This includes those generated by transgenic approaches, gene editing, chemical/radiation-based mutagenesis and hybridization. For transgenic lines, describe the transformation method, the number of independent lines analyzed and the generation upon which experiments were performed. For gene-edited lines, describe the editor used, the endogenous sequence targeted for editing, the targeting guide RNA sequence (if applicable) and how the editor was applied.*
Authentication	*Describe any authentication procedures for each seed stock used or novel genotype generated. Describe any experiments used to assess the effect of a mutation and, where applicable, how potential secondary effects (e.g. second site T-DNA insertions, mosiacism, off-target gene editing) were examined.*
