# Supplementary material for: Isthmin-2 is a first-trimester predictor of preeclampsia and fetal growth restriction
Source: Nat Med. 2026 Sep 1;32(9):3351–8. doi: 10.1038/s41591-026-04573-6 (PMC13577898; doi:10.1038/s41591-026-04573-6)
Supplement: Supplementary file 2 [file 41591_2026_4573_MOESM2_ESM.pdf]

nature portfolio

Peer Review File

Isthmin-2 is a first trimester predictor of preeclampsia and fetal growth restriction

Corresponding Author: Professor D. Stephen Charnock-Jones

This file contains all reviewer reports in order by version, followed by all author rebuttals in order by version.

Version 0:

Reviewer comments:

Reviewer #2

(Remarks to the Author)
In this paper By Miao and coworkers, the authors pinpoint a novel serum protein associated with major placental disease, this factor having not yet been identified before in this situation.

Bsaically the results are strong and straightforwardly presented, with quite large cohorts, and state-of-the art tools (organoids, RNA-seq).

The protein ISM2 was not identified previously in this context, despite a report mentioning the expression of the gene encoding Isthmin2 in the placenta, and its expressional decrease in preeclampsia (Martinez et al, Helyion 2020, ref 17 of the manuscript). This is not confirmed in other studies, since in a reference Hypertension paper (Leavey, Cox and Bainbridge, 2016), the analysis of the numerous cases did not reveal a significant expressional alteration of ISM2 in the placenta.

By all means, the study presented here is based upon SERUM components and addressed the different trimesters of pregnancy, with a technique that was not used before in a similar context which might explain why ISM2 was found de novo, and from a quite large cohorts at specific terms.

A very strong point is the validation on another large additional cohort of more than 2500 patients.

The technique Olink was performed by the society, leading to have the relative concentration of 2904 proteins out of 2944 potentially detected. The data demonstrate that ISM2 was the most down-regulated factor in a composite measure of the differences. Interestingly, other well-known circulating factors were detected such as sFLT1, LIFR or NOS2, sustaining the reliability of the results. The protein was found decreased in pathologies up to 28 weeks of gestation, and increased on the contrary in 36 weeks sera.
In my opinion, the authors made a very important discovery, adding a major protein to what is currently used to test for preeclampsia, essentially the sFLT1/PLGF ratio.

1. One question is that in Figure 1F, PIGF is not present while it is found in Supplementary Table 4 and 5. Is it because ony the factors found in all the conditions were presented there? But in Table sS2 there are also proteins that are not in Figure 1f, such as HMOX1 or SERPINB9 for instance, is it because there are extremely far in other pathological situations? It would then mean that specificities of each situation (isolated PE, PE+FGR, Isolated FGR), are quite important. It could be something worth discussing.

2. The ROC curves are interesting, but there is no information of ROC AUC when several markers are combined in terms of predicting disease including ISM2. The striking observation that the difference is higher early would potentially mean that ISM2 is better than everything we currently measure in patient plasmas. Especially the difference at 12 weeks, is compatible

with aspirin treatment, that to be efficient has to be administered before 16 weeks. It was a bit surprising not to have mention of this point by the authors.

To try to get insights into the function of ISM2 in context, the authors used trophoblast stem cells and trophoblast organoids. In organoids, ISM2 was colocalized with HLA-G. KD of ISM2 prevented the differentiation into EVT. Therefore, the role of ISM2 in EVT differentiation is well shown by the authors and EVT outgrowth from organoids were drastically reduced. The authors showed wound repair experiments with ISM2 inhibition in JEG3 and overexpression in HEK293, as well as conditioned medium from these cells.

3.Was the basal level of ISM2 in JEG-3 too high to envisage overexpressing it in this more relevant cell model to see whether the repair is accelerated?

(Remarks on code availability)

Reviewer #3

(Remarks to the Author)
Thank you for inviting me to review this manuscript, it was a very enjoyable read. The study identifies a potential new biomarker and therapeutic target for pregnancies complicated by PE and/or FGR and begins to unravel some of ISM2's mechanism of action. The findings are highly novel. The study design is well thought out, utilising 2 large pregnancy cohorts as well as a variety of in vitro models. Overall, I think the study is sound, but I would like to see an additional assessment of ISM2 in early vs late preeclampsia and some further interpretation of some of the mechanistic data.

Specific comments:
1. The authors have used the 2013 ACOG guidelines to define PE which is probably necessary given when the cohort was recruited. It is important however to sub-classify these patients as early vs. late onset PE given that the focus of this study is more relevant to the pathogenesis of early-onset PE. From Suppl. Table it appears that most are late-onset PE, which is considered more of a maternal rather placental disease. How do the authors reconcile this, given their hypothesis?

2. Is there a possibility that the cause of the lower ISM2 levels is due to fewer EVTs rather than a downregulation in expression? Alternatively, could reduced ISM2 expression impair EVT differentiation as is shown in your organoid model? It is well described that EVT invasion is impaired in PE and FGR placentation, what is known about cell numbers in vivo and what was the impact of ISM2 (knockdown or overexpression) in your in vitro studies on cell viability/proliferation? This is an important aspect that is missing. Without proliferation data, it is unclear whether the changes in migration are due solely to migration or also due to changes in cell number. Examining Invasion specifically would also be beneficial to further our understanding of the function of ISM2.

3. This study begins to examine the potential mechanism of action of ISM2 in the placenta by examining the effect of ISM2 knockdown on the transcriptome. The authors identified a large number of genes that were differentially expressed and identified 12 pathways. It appears that most of the pathways were not related to cell migration/invasion specifically and the pathways identified were not discussed. Further discussion of this data and potential mechanisms should be incorporated into the discussion. At present, there is some discussion of the potential for interaction with integrins, were integrins among the DEGs?

Minor comments:
1. Suggest using "is associated with later development of" rather than "predispose to" in title as cause and effect was not shown.
2. Abstract- specify size of cohorts, "large" is ambiguous
2. Line 143, please specify the 4 protein predictors here.
3. Fig. 3, please include scale bars in b and e
4. Discussion, lines 266-270. This final statement regarding autoantibodies to ISM2 is highly speculative, suggest removing.
5. Can you please include median (IQR) data for birth weight centile, BP, and protein/creatinine for each outcome group in Suppl. Tables 1 and 6. The proportion with PTB should also be included.
6. Were there any exclusion criteria for the groups, for example, GDM? These should also be stated in the methods.
7. How specific is the ISM2 Antibody that is used for IF? Does it cross-react with ISM1?
8. Why use Jeg-3 cells (choriocarcinoma) and not a first trimester EVT cell line, such as HTR-8/SVneo cells?
9. Please provide representative full-length blots for western blot data
10. What is the structure of organoids (inside out?), can the authors provide more detail about this in the methods/results.

(Remarks on code availability)

Version 1:

Reviewer comments:

Reviewer #2

(Remarks to the Author)
The authors responded extensively and in detail to my remarks about the first version.
It was very good, it is now excellent.

(Remarks on code availability)

Reviewer #3

(Remarks to the Author)
Thank you for making significant changes to the manuscript. I believe it is much improved and have no further recommendations.

(Remarks on code availability)
unable to view

Open Access This Peer Review File is licensed under a Creative Commons Attribution 4.0 International License, which permits use, sharing, adaptation, distribution and reproduction in any medium or format, as long as you give appropriate credit to the original author(s) and the source, provide a link to the Creative Commons license, and indicate if changes were made.
In cases where reviewers are anonymous, credit should be given to 'Anonymous Referee' and the source.
The images or other third party material in this Peer Review File are included in the article's Creative Commons license, unless indicated otherwise in a credit line to the material. If material is not included in the article's Creative Commons license and your intended use is not permitted by statutory regulation or exceeds the permitted use, you will need to obtain permission directly from the copyright holder.
To view a copy of this license, visit https://creativecommons.org/licenses/by/4.0/

Response to reviewers' comments

NMED-A145417-T

Editor's comments

#	Comment	Response
1	It would be essential that you provide validation in an independent, external cohort, ideally from a different demographic, even if from a historical database that can be interrogated, to confirm reproducibility of Isthmin-2 for preeclampsia/FGR in early pregnancy.	We have now additionally validated ISM2 in the IMPACT cohort (PMID 32967865), a multi-centre prospective cohort study of 12,473 pregnant women conducted in Sweden from 2018-2022. We measured ISM2 and PlGF in first trimester blood samples from 144 women of mixed parity with PE complicated by preterm birth, FGR, or both preterm birth and FGR. These women were compared with 256 control women of mixed parity, who did not experience PE and delivered a baby with a birth weight >10th percentile. The AUC for ISM2 was 0.709 (95% CI = 0.655 to 0.764) in the IMPACT cohort, which was very similar to the AUC for the composite outcome in POPS. The association with ISM2 in the IMPACT cohort was highly statistically significant ($P=2.62 \times 10^{-12}$), and the AUC for ISM2 in the IMPACT cohort was statistically significantly greater than the AUC for PlGF (AUC =0.645, 95% CI = 0.587 to 0.702, p for comparison with ISM2 [DeLong test] =0.012).<br><br>We have added a scatter plot of the raw data from IMPACT (Fig 2k) and the ROC curves for ISM2 and PlGF in the IMPACT cohort (Fig. 2l).<br><br>We have added the following text to the manuscript:

		"We performed a further external validation using data and samples from the IMPACT study[15], a Swedish prospective multicentre cohort of mixed parity which obtained blood between 11wkGA and 14wkGA. We measured first trimester levels of ISM2 and PlGF using a targeted proximity extension assay (Olink Focus panel), comparing pregnancies where the mother subsequently experienced preeclampsia complicated by either preterm birth or FGR with a randomly selected subset of controls (see Supplementary Table 8 for demographic details). Gestational age adjusted z scores of ISM2 were significantly lower among cases (t-test, $p = 2.62 \times 10^{-12}$) (Fig 2k) and ISM2 had a statistically significantly higher AUC than PlGF (Fig 2l)." Lines 165-173.
2	Benchmarking would be essential.	We have compared the predictive ability of ISM2 measured in the first trimester with the predictive ability of five existing placental biomarkers, measured using a clinical grade analysis platform. The discrimination achieved by ISM2 was statistically significantly greater than achieved by any of the five proteins.<br><br>We have added the benchmarking of ISM2 against the panel of five existing biomarkers to Fig 2 (panel c) and we have also included benchmarking against PlGF (the leading existing first trimester biomarker) in the POPS2 and IMPACT cohorts (Supplementary Fig. 3 and Fig. 2l respectively).<br><br>We have added the following text to the manuscript:<br>"When we benchmarked the association against existing biomarkers, the area under the receiver operating characteristic curve (AUC, a clinical measure of prognostic

		discrimination) of the composite outcome was statistically significantly higher for ISM2 measured using the Olink assay than for any of five placental biomarkers measured in the same samples at 12wkGA using a clinical analysis platform (Fig. 2c)." Lines 113-117.<br>"We compared the performance of ISM2 to PlGF in the POPS2 cohort (DeLong test) and ISM2 performed significantly better (Supplementary Fig. 3)". Lines 164-165<br>" ....and ISM2 had a statistically significantly higher AUC than PlGF (Fig 2l)". Lines 173-174
3	Ideally, the protein should be measured in the full cohorts, not only on a randomly selected fraction of them. Note that at present, the number of cases and controls is not clear from the main manuscript, but only referred to in suppl tables 1 and 6.	We have clarified the number of cases and controls in all experiments. In relation to analysing samples from the whole cohort, this would be extremely expensive and time consuming and would add virtually nothing to the analysis. The outcomes studied in our analysis are the most clinically significant manifestations of PE and FGR. Consequently, they are relatively uncommon. If you consider a disease that affects 2% (ie 1 in 50) of the population, analysing the entire cohort results in a ratio of 49 controls to each case. There is no material gain in the statistical precision of estimates by having such a high ratio of controls to cases. This is the rationale behind using a case cohort design. The strengths of this study design are explained in detail elsewhere:<br>PMID 35383643, and this review specifically addresses the utility of this study design when using expensive assays. We have added a citation to a study which uses this design as an example. We have also added a citation to a paper describing this approach in a cohort of 29,333 participants<br>(PMID 35403197). If there is remaining uncertainty, we

		recommend expert statistical review as we are confident that the experimental design is not merely adequate, but optimal.<br><br>We have added the following text to the manuscript: "The advantages of a case-cohort design have been described in detail elsewhere[12] and the large (29,333 participant) EPIC-CVD study exemplifies such a design[13]". Lines 73-74

Reviewer #2 (Remarks to the Author)

#	Comment	Response
2.1	In this paper By Miao and coworkers, the authors pinpoint a novel serum protein associated with major placental disease, this factor having not yet been identified before in this situation. Basically the results are strong and straightforwardly presented, with quite large cohorts, and state-of-the art tools (organoids, RNA-seq). The protein ISM2 was not identified previously in this context, despite a report mentioning the expression of the gene encoding Isthmin2 in the placenta, and its expressional decrease in preeclampsia (Martinez et al, Helyion 2020, ref 17 of the manuscript). This is not confirmed in other studies, since in a reference Hypertension paper (Leavey, Cox and Bainbridge, 2016), the analysis of the numerous cases did not reveal a significant expressional alteration of ISM2 in the placenta.<br>By all means, the study presented here is based upon SERUM components and addressed the different trimesters of pregnancy, with a technique that was not used before in a	We are grateful to the reviewer for their positive comments.

	similar context which might explain why ISM2 was found de novo, and from a quite large cohorts at specific terms. A very strong point is the validation on another large additional cohort of more than 2500 patients. The technique Olink was performed by the society, leading to have the relative concentration of 2904 proteins out of 2944 potentially detected. The data demonstrate that ISM2 was the most down-regulated factor in a composite measure of the differences. Interestingly, other well-known circulating factors were detected such as sFLT1, LIFR or NOS2, sustaining the reliability of the results. The protein was found decreased in pathologies up to 28 weeks of gestation, and increased on the contrary in 36 weeks sera. In my opinion, the authors made a very important discovery, adding a major protein to what is currently used to test for preeclampsia, essentially the sFLT1/PLGF ratio.	
2.2	One question is that in Figure 1F, PlGF is not present while it is found in Supplementary Table 4 and 5. Is it because any the factors found in all the conditions were presented there? But in Table sS2 there are also proteins that are not in Figure 1f, such as HMOX1 or SERPINB9 for instance, is it because there are extremely far in other pathological situations? It would then mean that specificities of each situation (isolated PE, PE+FGR, Isolated FGR), are quite important. It could be something worth discussing.	Figure 1F presents data which reflects the overall strength of association between a given protein and **all** the outcomes studied. Hence, a protein might be strongly associated with one of the outcomes but if it was more weakly associated with the other outcomes it would not be listed in the top 10 proteins in Figure 1F. As noted by the referee, we have included all of the data in our supplementary tables, hence the interested reader is able to explore such details of the associations.<br><br>The opening paragraph of the discussion states that ISM2 was the only protein of the 2904 proteins to be strongly associated with both PE and FGR. While there are other proteins which

		are also associated with either PE or FGR, none were more strongly associated with these outcomes than ISM2. Hence, we have not added to the discussion and are happy that the interested reader has access to all the DAPs in the supplementary tables.
2.3	The ROC curves are interesting, but there is no information of ROC AUC when several markers are combined in terms of predicting disease including ISM2. The striking observation that the difference is higher early would potentially mean that ISM2 is better than everything we currently measure in patient plasmas. Especially the difference at 12 weeks, is compatible with aspirin treatment, that to be efficient has to be administered before 16 weeks. It was a bit surprising not to have mention of this point by the authors.	We agree that it would have been surprising not to mention this. However, lines 140-149 in the original MS make it clear that ISM2 on its own was a better predictor or as good a predictor of later complications than combinations of proteins. Moreover, we can be confident in this conclusion as it was better across all of 19 comparisons of different modelling approaches (statistical, machine learning and AI) as documented in Supplementary Table 9.<br><br>This element of the analysis is described in lines 609 to 623 in the revised MS.
2.4	To try to get insights into the function of ISM2 in context, the authors used trophoblast stem cells and trophoblast organoids. In organoids, ISM2 was colocalized with HLA-G. KD of ISM2 prevented the differentiation into EVT. Therefore, the role of ISM2 in EVT differentiation is well shown by the authors and EVT outgrowth from organoids were drastically reduced. The authors showed wound repair experiments with ISM2 inhibition in JEG3 and overexpression in HEK293, as well as conditioned medium from these cells.<br><br>Was the basal level of ISM2 in JEG-3 too high to envisage overexpressing it in this more relevant cell model to see whether the repair is accelerated?	We confined our over-expression experiments to cells which did not constitutively express ISM2. Indeed, one of the most remarkable findings in the paper is that ISM2 expression enhanced cell migration in a kidney derived cell line. We do not think that over-expressing the protein in a cell type which already has a high level of expression is a particularly informative experiment. If over-expression had no effect, it could be that ISM2 is being expressed at a level which exceeds its maximum effect on cell migration. Hence, a negative result would not shed light on the importance of the protein in this cell type. We believe knocking the protein down where it is expressed and expressing the protein where it is not are the logical and most relevant experiments.

		Additionally, Reviewer 3 (minor comment 8) raised concerns about the use of JEG3 choriocarcinoma cell lines to model the EVT invasion. Hence, we have replaced the data using the suboptimal JEG3 cell line and have now performed directed invasion assays using a trophoblast stem cell-derived EVT cells (PMID: 38346993). Please refer to the response to Reviewer 3 comment below (major comment 3 and minor comment 8).

Reviewer #3 (Remarks to the Author)

#	Comment	Response
3.1	Thank you for inviting me to review this manuscript, it was a very enjoyable read. The study identifies a potential new biomarker and therapeutic target for pregnancies complicated by PE and/or FGR and begins to unravel some of ISM2's mechanism of action. The findings are highly novel. The study design is well thought out, utilising 2 large pregnancy cohorts as well as a variety of in vitro models. Overall, I think the study is sound, but I would like to see an additional assessment of ISM2 in early vs late preeclampsia and some further interpretation of some of the mechanistic data.	We are grateful to the reviewer for their positive comments, and we note that both reviewers comment very positively on the novelty and importance of this work.
3.2	The authors have used the 2013 ACOG guidelines to define PE which is probably necessary given when the cohort was recruited. It is important however to sub-classify these patients as early vs. late onset PE given that the focus of this study is more relevant to the pathogenesis of early-onset PE. From Suppl. Table it appears that most are late-onset PE,	We are grateful to the reviewer for their comment. We have now added separate analyses of the composite outcome by whether the delivery was preterm or at term. This analysis demonstrates that ISM2 was more strongly associated with complications leading to preterm birth. This is consistent with the view, which the reviewer alludes to, namely, that failure of

	which is considered more of a maternal rather placental disease. How do the authors reconcile this, given their hypothesis?	trophoblast invasion is particularly strongly associated with placental complications resulting in preterm birth. Hence, our novel findings are consistent with a large body of evidence.<br><br>We have added ROC curves for the composite outcome, separated into cases resulting in preterm or term birth (Fig. 2d).<br><br>We have added the following text to the manuscript: "The AUC for ISM2 at 12wkGA was higher for cases of the composite outcome resulting in preterm birth compared with term birth (Fig. 2d)". Lines 117 to 118.
3.3	Is there a possibility that the cause of the lower ISM2 levels is due to fewer EVTs rather than a downregulation in expression? Alternatively, could reduced ISM2 expression impair EVT differentiation as is shown in your organoid model? It is well described that EVT invasion is impaired in PE and FGR placentation, what is known about cell numbers in vivo and what was the impact of ISM2 (knockdown or overexpression) in your in vitro studies on cell viability/proliferation? This is an important aspect that is missing. Without proliferation data, it is unclear whether the changes in migration are due solely to migration or also due to changes in cell number. Examining Invasion specifically would also be beneficial to further our understanding of the function of ISM2.	We are grateful to the reviewer for raising this important point. Previous studies demonstrate reduced numbers of EVTs within the placental bed (decidua and myometrium) in pregnancies complicated by preeclampsia and fetal growth restriction (PMIDs: 12848643 and 911717). However, it is unclear whether the reduced EVT numbers in the placental bed are due to the decreased number of EVTs to begin with (i.e. a differentiation defect) or reduced capacity of the differentiated EVTs to invade deeply into the endometrium (i.e. an invasion defect). It is likely a combination of both these factors as there is evidence to support both these hypotheses (PMIDs: 35168169, 12848643 and 911717). Given that EVTs lose their proliferative capacity as they migrate from the cytotrophoblast cell columns (PMID: 3225818), any changes in EVT numbers within the placental bed are likely a result of: (i) reduced differentiation of cytotrophoblasts into EVTs, (ii) impaired EVT migration and/or (iii) reduced numbers

		of the progenitor cytotrophoblasts from which EVTs arise. We have now determined whether ISM2-knockdown influences each of these 3 processes.<br><br>(a) ISM2-KD and effects on hTSC differentiation into EVTs.<br>We have performed additional RNA-sequencing analyses to determine whether ISM2 predominantly affects trophoblasts in the EVT state. We performed RNA-sequencing of trophoblast stem cells stably transduced with Scr-shRNA or ISM2-shRNA and compared the number of differentially expressed genes (DEGs) in the following cell types: (1) cells maintained in their stem state as hTSCs, (2) cells differentiated into syncytiotrophoblast (STB), and (3) cells differentiated into EVTs. This last group was further sub-divided based on the stage of differentiation: mid or terminal. The summary of this analysis is as follows: (i) The number of DEGS associated with ISM2 knockdown was greater in EVT compared to STB or hTSCs. The number of DEGs were: hTSCs = 1034, STB = 679, EVT mid = 696 and EVT terminal = 1113. (ii) ISM2-KD in both EVT-mid and EVT-terminal cells resulted in DEGs that were negatively enriched for the Descartes term “Extravillous_Trophoblast”. In contrast, the DEGs resulting from ISM2-KD in the STB or hTSC-state were not enriched for the equivalent Descartes terms “Syncytiotrophoblast” or “Cytotrophoblast”, respectively. These findings strongly suggest that the effects of ISM2-KD are amplified if cells are in the EVT stage, indicating a defect in the differentiation program.

		We have added the new data to Fig. 3g and Fig S6 (panels a, b, c).<br><br>We have added the following text to the manuscript:<br>"We performed RNA-seq analysis comparing *ISM2* knockdown and control trophoblasts in either their stem-state (hTSCs), or following differentiation into STB and EVTs (at mid-differentiation point – referred to as EVT mid) or into terminally differentiated EVTs (referred to as EVT terminal). This analysis identified 1,034 differentially expressed genes (DEGs) in hTSCs, 679 DEGs in STB, 696 DEGs in EVT mid and 1,113 DEGs in EVT terminal (edgeR; adjusted p value <0.05; absolute fold change >2; Fig. 3g; Supplementary Fig. 6a – 6c; Supplementary Tables 11 - 14)." Lines 231 to 236<br><br>"Cell-type signature analysis in mid-differentiation EVTs also demonstrated significant enrichment with fetal EVT in down-regulated genes, albeit to a lesser extent (adjusted p value = 0.01, Supplementary Fig. 6d). However, the same analysis in hTSCs and STB did not reveal any significant enrichments with their corresponding cell type (Supplementary Fig. 7d and 7e). In addition to the down-regulated genes, there were 530, 449, 302 and 772 up-regulated genes in hTSCs, STB, mid-differentiation EVTs and terminally differentiated EVTs respectively (edgeR, adjusted p value <0.05; absolute fold change >2, Supplementary Tables 11-14). We then focused our analysis on terminally differentiated EVTs since they demonstrated the largest number of DEGs." Lines 247 to 255)

		(b) Effects of ISM2-knockdown on EVT migration.<br>We previously used JEG3 cells, a choriocarcinoma cell line which exhibits EVT-like phenotype and showed reduced migration (wound healing) following ISM2-knockdown. We selected this cell model because EVTs differentiated from trophoblast stem cells do not form a confluent layer, hence wound healing assays cannot be performed using this cell type. Differentiated EVTs also do not migrate across transwells and therefore do not allow transwell migration assays to be performed. These constraints led us to study JEG3 cells. Nonetheless we agree with this reviewer that JEG3 are not the ideal model (noted as a minor comment 8 below). A more recently published method (PMID: 38346993) allows us to quantify EVT invasion into Matrigel in 2D cultures using EVT differentiated from hTSCs. We now provide additional data using this method. These additional assays demonstrate that ISM2-KD impairs EVT invasion. We have now removed the cell migration data using JEG3 cells and replaced it with the newer method for quantifying EVT migration.<br><br>We have added the data to Fig. 4 (panel b, replacing the JEG3 wound healing assay).<br><br>We have added the following text to the manuscript:<br>“Consistent with the impairment in EVT phenotype, *ISM2* knockdown also reduced the invasive capacity of EVT into a Matrigel matrix (Fig. 4b).” Lines 265-266<br><br>(c) Effects of ISM2-knockdown on hTSC proliferation and viability.

		We have now carried out MTT cell viability assays which show no significant changes between Scr-shRNA and ISM2-shRNA. Cell proliferation assays were previously shown in Supplementary (now Figure S5b) – there were no differences in control versus ISM2-knockdown. These findings strongly suggest that the effects of ISM2-knockdown on EVT differentiation and invasion are not due alterations in cell viability or proliferation.<br><br>We have added the MTT assay data to Figure S5 (panel c) and the following text to the manuscript:<br>“ISM2 knockdown did not affect hTSC proliferation, cell viability or differentiation into STB lineage (Supplementary Figs. 5b-d).” Lines 202 to 204.
	This study begins to examine the potential mechanism of action of ISM2 in the placenta by examining the effect of ISM2 knockdown on the transcriptome. The authors identified a large number of genes that were differentially expressed and identified 12 pathways. It appears that most of the pathways were not related to cell migration/invasion specifically and the pathways identified were not discussed. Further discussion of this data and potential mechanisms should be incorporated into the discussion. At present, there is some discussion of the potential for interaction with integrins, were integrins among the DEGs?	We have now added GSEA of the disease associated proteins for PE and FGR. This demonstrates that PE, FGR and the composite outcome were all associated with interferon alpha and gamma pathways. These pathways were also identified in the analysis of differentially expressed genes following ISM2 knockdown in EVT. These interferons are known to be important in the function of NK cells, and uterine NK cells are thought to have an important role in regulating trophoblast invasion. We now discuss these analyses. Moreover, we now provide additional evidence supporting a potential role for integrins and have expanded on the discussion in relation to mechanism. *In vivo*, cytotrophoblast differentiation into invasive extravillous trophoblasts is accompanied by spatial and temporal changes in the integrin repertoire – referred to as “integrin switching” (PMID: 7529679). For example, α1β1 and α5β1 integrins are upregulated as cytotrophoblasts

		differentiate from hTSC into EVTs. We now provide data showing the effect of ISM2 knockdown on the expression of these genes (Supplementary Tables 11-14). RNA-seq analysis of hTSCs and EVTs (mid and terminally differentiated) and ISM2-KD indicated a role for ISM2 in this process: the normal upregulation of the genes encoding the α1 and α5 subunits on differentiation from hTSC to EVT was blunted by ISM2 knockdown (shown in Supplementary Figures 7a-c).<br><br>We have added the new data to Supplementary Figures 4 and 7a-c, and Supplementary Tables 11-14.<br><br>We have added the following text to the manuscript: "Moreover, integrins are associated with trophoblast invasion *in vivo* [20], and *ITGA5* was decreased in hTSCs and mid-differentiation EVTs whereas *ITGA1* was decreased in terminally differentiated EVTs following *ISM2* knockdown (Supplementary Fig. 7a – 7c)." Lines 242-245<br><br>"For example, cytotrophoblast differentiation into invasive EVTs is accompanied by spatial and temporal changes in the integrin repertoire *in vivo*, a process referred to as "integrin switching". Consistent with this, upregulation of integrins *ITGA5* and *ITGA1* with hTSCs differentiating into EVTs was prevented by *ISM2* knockdown." Lines 344-348.

Minor comments

#	Comment	Response
	Suggest using "is associated with later development of" rather than "predispose to" in title as cause and effect was not shown.	In order to keep the title as brief as possible we have replaced “predispose to” with “precede”.
3m.1	Abstract- specify size of cohorts, “large” is ambiguous	We have deleted the word “large” rather than lengthen the abstract any further.
3m.2	Line 143, please specify the 4 protein predictors here	Done
3m.3	Fig. 3, please include scale bars in b and e	Done
3m.4	Discussion, lines 266-270. This final statement regarding autoantibodies to ISM2 is highly speculative, suggest removing	We have qualified this section but we feel that it is worth considering different mechanisms which might lead to reduced ISM2.
3m.5	Can you please include median (IQR) data for birth weight centile, BP, and protein/creatinine for each outcome group in Suppl. Tables 1 and 6. The proportion with PTB should also be included.	We have added the data requested to new Supplementary Tables 1a-1d and revised Supplementary Table 6. Note: the protein:creatinine ratio was not used in the POPS cohort, as it was conducted in 2008-2103. We have included the measures of proteinuria which were employed at the time.
3m.6	Were there any exclusion criteria for the groups, for example, GDM? These should also be stated in the methods	The only group excluded were cases of stillbirth.<br>We have added this information to the manuscript (lines 482-483).
3m.7	How specific is the ISM2 Antibody that is used for IF? Does it cross-react with ISM1?	Our data confirm the specificity of the ISM2 assay used here. ISM2 was undetectable by the Olink proximity extension assay in EVTs following ISM2 knockdown (Fig. 3d). Transfection of cells that do not express ISM2 (HEK293) resulted in robust ISM2 expression that was readily detectable in the culture media by the Olink assay (Supplementary Fig. 8). ISM1, which is measured on the Olink Explore panel showed no disease association.

		The ISM2 antibody used for immunofluorescence and immunoblotting (Proteintech; #21540-1-AP) was raised against 71-420aa of ISM2. Alignment of this region with human ISM1 shows only 37% identity. siRNA-mediated ISM2 knockdown abolished the immunoblotting signal. This is consistent with results obtained with the Olink assay (Supplementary Fig 8).<br><br>We have added the following text to the manuscript:<br>"Our data confirm the specificity of the ISM2 assay used here. ISM2 was undetectable by the Olink Proximity extension assay in EVTs following shRNA-mediated knockdown (Fig. 3d). Transfection of cells that do not express ISM2 (HEK293) resulted in robust ISM2 expression that was readily detectable by the Olink assay (Supplementary Fig. 8). ISM1, which is measured by the Olink Explore panel showed no disease association." Lines 310-314.
3m.8	Why use Jeg-3 cells (choriocarcinoma) and not a first trimester EVT cell line, such as HTR-8/SVneo cells?	Neither JEG-3 nor HTR8/Svneo cells are ideal models for EVT since JEG-3 is a choriocarcinoma cell line with EVT features whereas HTR-8/SVneo are transformed cells from first trimester trophoblast outgrowths. Additionally, questions over the purity of HTR8/SVneo have been raised since they contain a mixture of both trophoblasts and mesenchymal cells (PMID: 28161053). However, as stated in response to comment 3.3 above, we now show directional invasion assays using trophoblast stem cell-derived EVTs and have removed the data related to JEG3 cells.
3m.9	Please provide representative full-length blots for western blot data	Full length blots are shown in Source data Figure 3c, and Source data supplementary Figure 8a

3m.10	What is the structure of organoids (inside out?), can the authors provide more detail about this in the methods/results	Trophoblast organoids were grown in the conventional inside-out (i.e. apical-in) with the STB layer on the inside and cytotrophoblast layer on the outside as described in Turco 2018, Li 2024 and Shannon 2024 (PMIDs: 38359834, 38237587, 30487605). EVT organoids cannot be grown in physiological polarity (i.e. with STB on the outside) since the outgrowth of EVTs require CTBs to be in contact with the Matrigel surrounding the organoids. Additionally, apical out organoids with STB on the outside do not form a complete syncytium, cannot be cultured for long periods in suspension culture and cannot differentiate into EVTs (PMIDs: 37676312, 38332125, 40129708).<br><br>We have added the following text to the manuscript: "Trophoblast organoids were grown in their standard conformation (apical in) with the STB facing the inside core and TSCs facing the Matrigel on the outside to allow EVTs to differentiate and migrate from the TSCs." Lines 654-656.
